# Local Multiset Dimension of Amalgamation Graphs

**DOI:** 10.12688/f1000research.128866.1

**Published:** 2023-01-24

**Authors:** Ridho Alfarisi, Liliek Susilowati, Dafik Dafik, Savari Prabhu

**Affiliations:** 1 Elementary School Teacher Education, Universitas Jember, Jember, East Java, 68121, Indonesia; 2 Mathematics, Universitas Airlangga, Surabaya, Surabaya, 68121, Indonesia; 3 Mathematics Education, Universitas Jember, Jember, East Java, 68121, Indonesia; 4 Department of Mathematics, Rajalakshmi Engineering College, Chennai, Tamil Nadu, 602105, India

**Keywords:** local m-resolving set; local multiset dimension; amalgamation graph.

## Abstract

**Background**: One of the topics of distance in graphs is the resolving set problem. Suppose the set *W* = { *s*_1_, *s*_2_, …, *s _k_*} ⊂ *V* ( *G*), the vertex representations of ∈ *V* ( *G*) is *r _m_* ( *x*| *W*) = { *d*( *x*, *s*_1_), *d*( *x*, *s*_2_), …, *d*( *x*, *s _k_*)}, where *d*( *x*, *s _i_*) is the length of the shortest path of the vertex *x* and the vertex in *W* together with their multiplicity. The set *W* is called a local *m*-resolving set of graphs *G* if *r _m_* ( *v*| *W*)≠ *r _m_* ( *u*| *W*) for *uv* ∈ *E*( *G*). The local *m*-resolving set having minimum cardinality is called the local multiset basis and its cardinality is called the local multiset dimension of *G*, denoted by *md _l_* ( *G*). Thus, if *G* has an infinite local multiset dimension and then we write ${\mathrm{md}}_{l}\left(G\right)=\infty$ .

**Methods**: This research is pure research with exploration design. There are several stages in this research, namely we choose the special graph which is operated by amalgamation and the set of vertices and edges of amalgamation of graphs; determine the set *W* ⊂ *V* ( *G*); determine the vertex representation of two adjacent vertices in *G*; and prove the theorem.

**Results**: The results of this research are an upper bound of local multiset dimension of the amalgamation of graphs namely *md _l_* ( *Amal*( *G*, *v*, *m*)) ≤ *m.* *md _l_* ( *G*) and their exact value of local multiset dimension of some families of graphs namely *md _l_* ( *Amal*( *P _n_*, *v*, *m*)) = 1, ${\mathrm{md}}_{l}\left(\text{Amal}\left(K_{n},v,m\right)\right)=\infty$ , *md _l_* ( *Amal*( *W _n_*, *v*, *m*)) = *m.* *md _l_* ( *W _n_*), *md _l_* ( *Amal*( *F _n_*, *v*, *m*)) = *m.* *md _l_* ( *F _n_*) for *d*( *v*) = *n*, ${\mathrm{md}}_{l}\left(\text{Amal}\left(F_{n},v,m\right)\right)=m.\left\lfloor \frac{n}{4}\right\rfloor$ .

**Conclusions**: We have found the upper bound of a local multiset dimension. There are some graphs which attain the upper bound of local multiset dimension namely wheel graphs

## Introduction

In this paper, we study the local multiset dimension of the amalgamation of graphs. One of the topics of distance in graphs is resolving set problems. This topic has many application in science and technology namely in the navigation of robots, chemistry structure, and computer sciences. The application of metric dimension in networks is the navigation of robots. Suppose, we represent a place as a vertex and a connection between place is represented by an edge. The minimum numbers of robots required to locate each vertex in the networks is part of the resolving set problems. More details of this application can be seen. ^1^

Several applications of resolving sets in chemistry are the substructures of a chemical compound which can be denoted by a set of functional groups. Moreover, in a chemical structure or molecular graph edges and vertices are known as bonds and atoms, respectively. Furthermore, the sub graphs are simply deliberated as substructures and functional groups. Now after altering the position of functional groups, the formed collections of compounds are distinguished as substructures being similar to each other. Later on using the method of traditional view, we can investigate if any two compounds hold the same functional group at the same point, while in drugs discovery comparative statements contributes a critical part to determine pharmacological activities related to the feature of compounds. ^2^

All graphs *G* are simple, finite, and connected. Given that the vertex set *V* ( *G*) and the edge set *E*( *G*), we write *G* = ( *V*, *E*). The distance of *u* and *v*, denoted by *d*( *u*, *v*) is the length of the shortest path of the vertices *u* to *v.* For the set *W* = { *s*_1_, *s*_2_, …, *s _k_*} ⊂ *V* ( *G*), the vertex representations of the vertex *x* to the set *W* is an ordered *k*-tuple, *r*( *x*| *W*) = ( *d*( *x*, *s*_1_), *d*( *x*, *s*_2_), …, *d*( *x*, *s _k_*)). The set *W* is called the resolving set of *G* if all vertices of *G* have different vertex representations. The resolving set having minimum cardinality is called basis and its cardinality is called metric dimension of *G*, denoted by *dim*( *G*). ^3^ Okamoto *et al.* ^4^ introduced the new variant of resolving set problems which is called local resolving set problems. In his paper, this notion is called a local multiset dimension of graphs *G.* The set *W* is called a local resolving set if for every *xy* ∈ *E*( *G*), *r*( *x*| *W*)≠ *r*( *y*| *W*). The local resolving set having minimum cardinality is called local basis and it’s cardinality is called local metric dimension of *G* and denoted by *ldim*( *G*).

Simanjuntak *et al*. ^5^ defined multiset dimension of graphs *G.* Suppose the set $W=\left\{s_{1},s_{2},\ldots ,s_{k}\right\}\subset V\left(G\right)$ , the vertex representations of a vertex *x* ∈ *V* ( *G*) to the set *W* is the multiset $r_{m}\left(x|W\right)=\left\{d\left(x,s_{1}\right),d\left(x,s_{2}\right),\ldots ,d\left(x,s_{k}\right)\right\}$ where *d*( *x*, *s _i_*) is the length of the shortest path of the vertex *x* and the vertex in *W* together with their multiplicities. The set *W* is called a *m*-resolving set if for every *xy* ∈ *E*( *G*), *r _m_* ( *x*| *W*)≠ *r _m_* ( *y*| *W*). If *G* has a *m*-resolving set then an m-resolving set having minimum cardinality is called a multiset basis and it’s cardinality is called the multiset dimension of graphs *G* and denoted by *md*( *G*); otherwise we say that *G* has an infinite multiset dimension and we write $\mathrm{md}\left(G\right)=\infty$ . Alfarisi *et al*. ^6^ studied the multiset dimension of almost hypercube graphs. Later, Alfarisi *et al.* ^7^ extended a new notion based on the multiset dimension of *G*, namely a local multiset dimension. Suppose the set $W=\left\{s_{1},s_{2},\ldots ,s_{k}\right\}\subset V\left(G\right)$ , the vertex representations of a vertex *x* ∈ *V* ( *G*) to the set *W* is *r _m_* ( *x*| *W*) = { *d*( *x*, *s*_1_), *d*( *x*, *s*_2_), …, *d*( *x*, *s _k_*)}. The set *W* is called a local *m*-resolving set of *G* if *r _m_* ( *v*| *W*)≠ *r _m_* ( *u*| *W*) for *uv* ∈ *E*( *G*). The local *m*-resolving set having minimum cardinality is called the local multiset basis and it’s cardinality is called the local multiset dimension, denoted by *md _l_* ( *G*): otherwise we say that *G* has an infinite local multiset dimension and we write ${\mathrm{md}}_{l}\left(G\right)=\infty$ .

**Figure 1.**
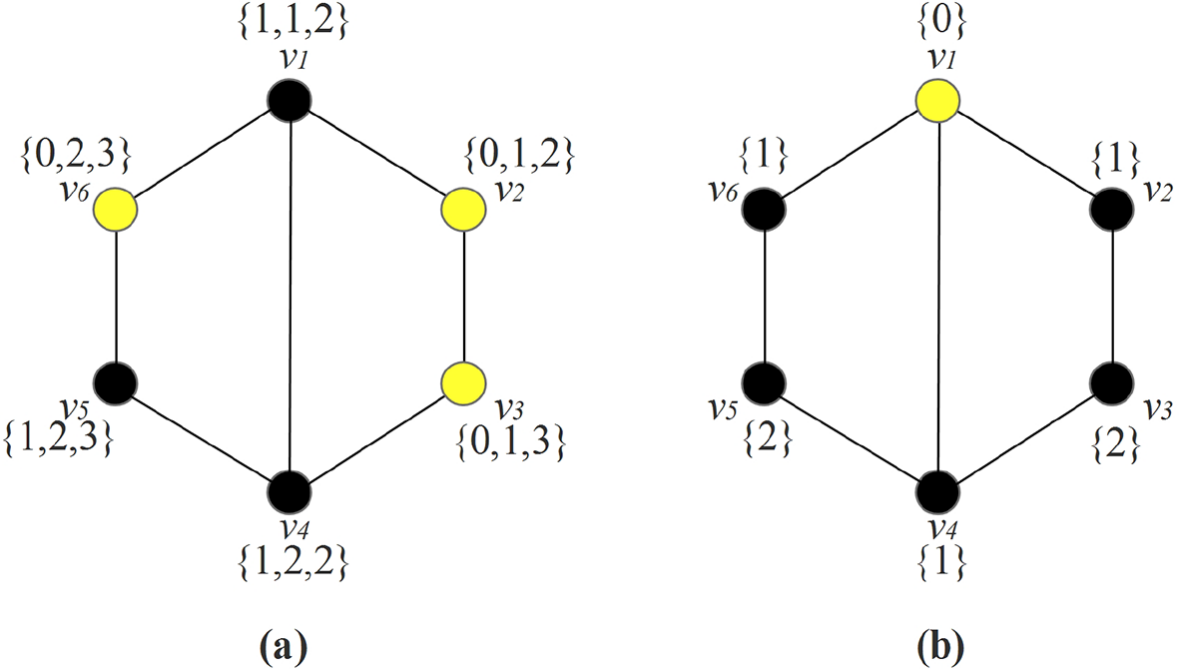
(a) A graph with multiset dimension 3; (b) A graph with local multiset dimension 1.

We illustrate this concept in Figure 1. In this case, the *m*-resolving set is *W* = { *v*_2_, *v*_3_, *v*_6_}, shown in Figure 1(a). The multiset dimension is *md*( *G*) = 3. The representations of *v* ∈ *V* ( *G*) with respect to *W* are all distinct. For the local multiset dimension, we only need to make sure the adjacent vertices have distinct representations. Thus, we could have the local *m*-resolving set *W* = { *v*_1_}, shown in Figure 1(b). Thus, the local multiset dimension is *μ _l_* ( *G*) = 1.

$$r\left(v_{1}|\Pi \right)=\left\{0\right\},\;r\left(v_{2}|\Pi \right)=\left\{1\right\},\;r\left(v_{3}|\Pi \right)=\left\{2\right\}$$

$$r\left(v_{4}|\Pi \right)=\left\{1\right\},\;r\left(v_{5}|\Pi \right)=\left\{2\right\},\;r\left(v_{6}|\Pi \right)=\left\{1\right\}$$

We have some results on the local multiset dimension of some known graphs namely path, star, tree, and cycle and also the local multiset dimension of graph operations namely, cartesian product, ^7^ *m*-shadow graph. ^8^ Adawiyah *et al.* ^9^ also studied local multiset dimension of unicyclic graphs. The followings are some results which is used for proving the new results in this study.

Lemma 0.1
*Let G be a connected graphs and W* ⊂ *V* ( *G*). *If W contains a resolving set of G*, *then W is a resolving set of G.* ^10^

Proposition 0.1
*A graph is bipartite if and only if it contains no odd cycle.* ^11^

Theorem 0.1
*The local multiset dimension of G is one if and only if G is a bipartite graph.* ^12^

Theorem 0.2
*If T is tree graph with order n*, *then md _l_* ( *T*) = 1. ^12^

Proposition 0.2
*Let K _n_ be a complete graph with n* ≥ 3, *we have* ${\mathrm{md}}_{l}\left(K_{n}\right)=\infty$ . ^6^

Definition 0.1
*Let* ( *G _i_*) *be a finite collection of graphs and each G _i_ has a fixed vertex v called a terminal. The amalgamation Amal* ( *G _i_*, *v*, *m*) *is formed by taking of all the G _i_ and identifying their terminal.*

**Figure 2.**
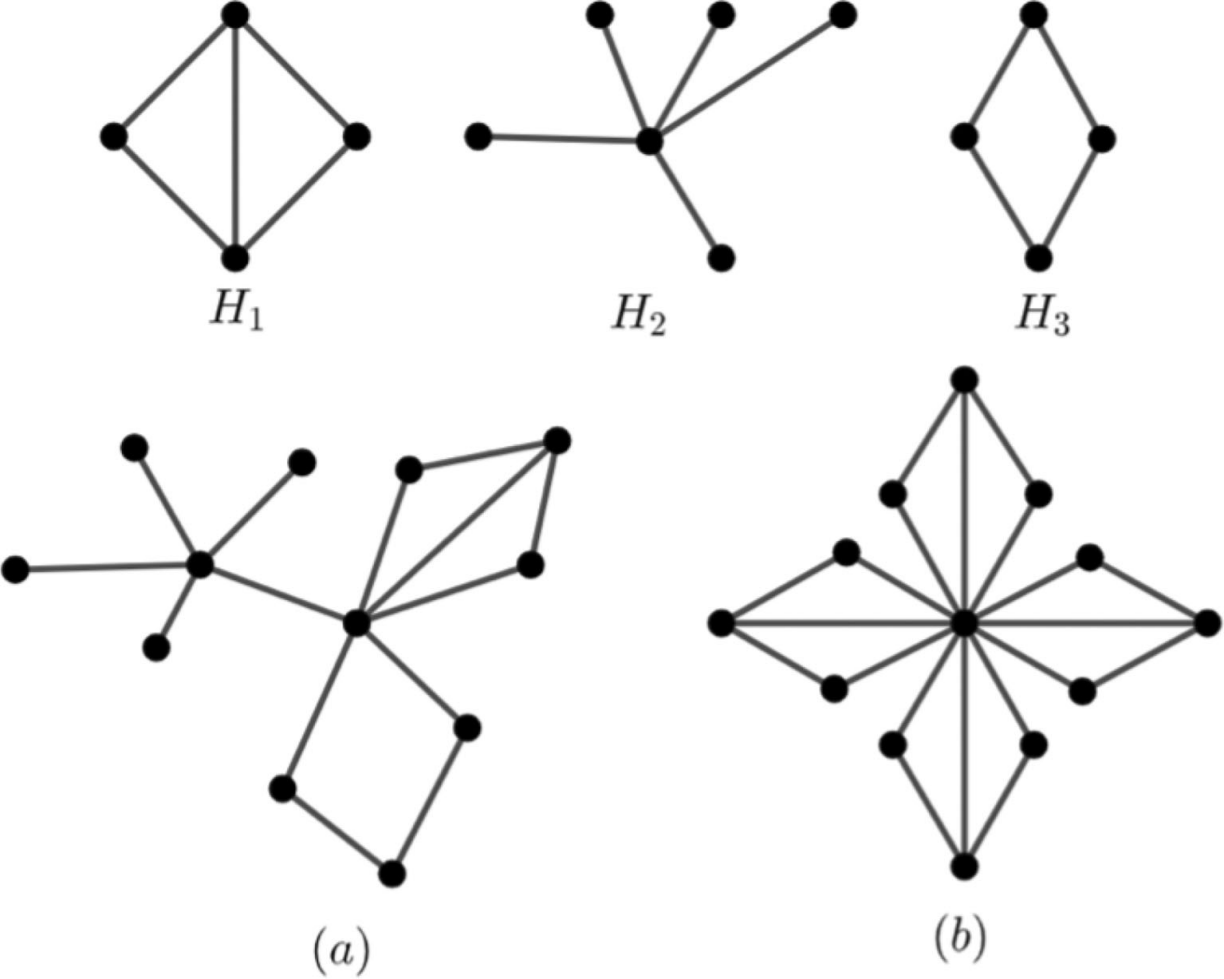
a) *Amal* ( *H _j_*, *v*_0, *j*_) with *j* = 1, 2, 3 and b) *Amal*( *H*_1_, *v*_0, 1_, 4).

Figure 2 is an example of an amalgamation graph with isomorphic and non-isomorphic graph.

## Methods

The nature of the methods follows an extrapolative design. There are several stages in this research as follows:

1. Choose the special graph which is operated by amalgamation;
2. Determining the set of vertices and edges of amalgamation of graphs;
3. Determining the set *W* ⊂ *V* ( *G*);
4. Determining the vertex representation of two adjacent vertices in *G*;
5. Proving the theorem.

**Figure 3.**
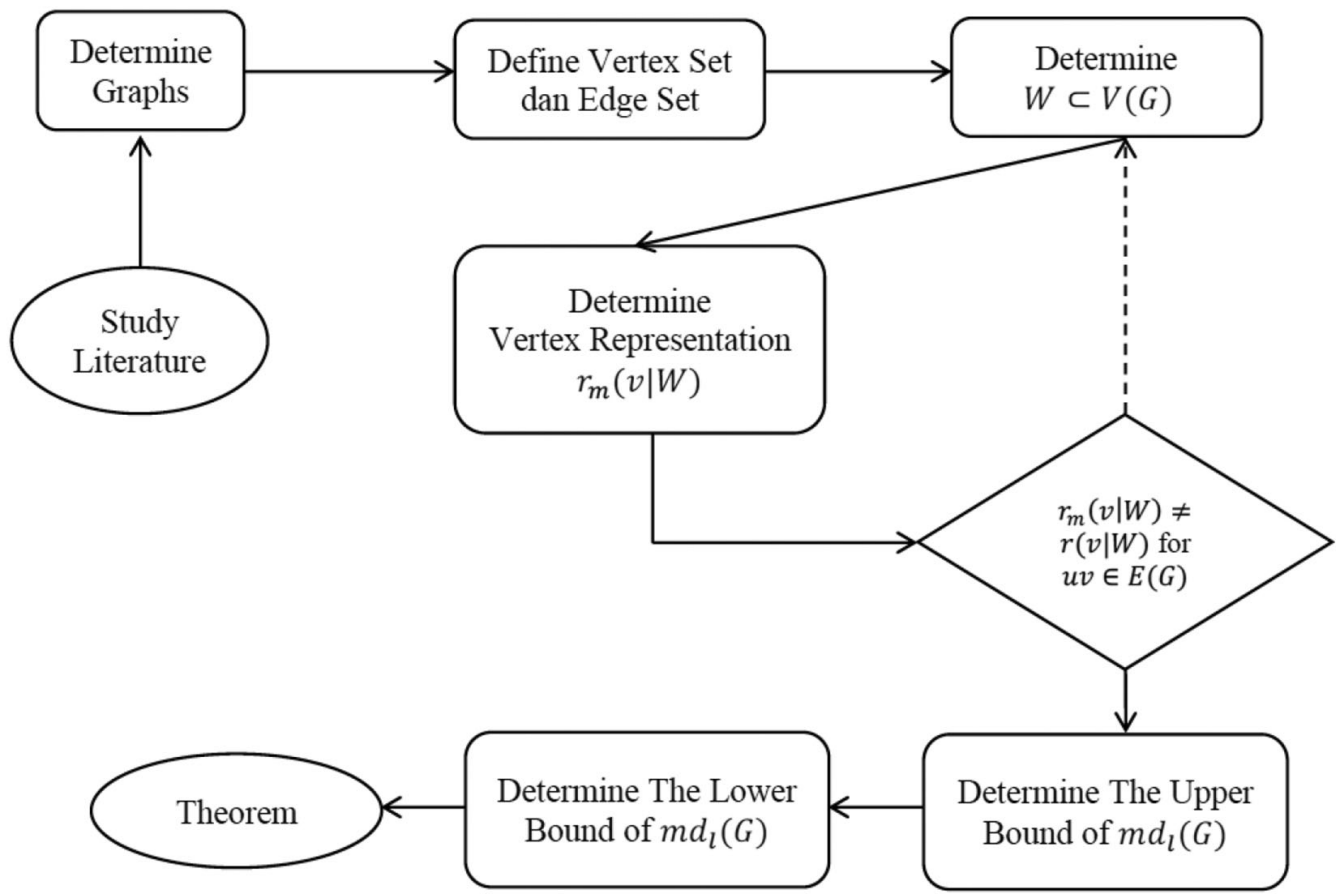
Flowchart of the extrapolate design of the study.

The flowchart of this method can be seen in Figure 3.

## Results

In this section, we investigated the local multiset dimension of graph amalgamation. We provide an upper bound of local multiset dimension of *Amal*( *G*, *v*, *m*) and we show that the upper bound is sharp. We also determined the exact value of the local multiset dimension of *Amal*( *G*, *v*, *m*) for some certain graphs namely path, complete graph, fan graph, and wheel graph. The following theorem provides a sharp upper bound of *Amal*( *G*, *v*, *m*).

Lemma 0.2
*Let m and n be two integers with m* ≥ 2 *and n* ≥ 3. *Let G be a connected graph of order n and v be a terminal vertex of G*, *then md _l_* ( *Amal*( *G*, *v*, *m*)) ≤ *m.* *md _l_* ( *G*)

Proof.
Let

$$V\left(\text{Amal}\left(G,v,m\right)\right)=\left\{u\right\}\cup \left\{u_{j,i};1\leq j\leq m,1\leq i\leq n-1\right\}$$

and

$$E\left(\text{Amal}\left(G,v,m\right)\right)=\overset{j=m}{\underset{j=1}{\cup }}E\left(G\right)$$

where *v* denote the identified vertex or terminal vertex.

We show that *md _l_* ( *Amal*( *G*, *v*, *m*)) ≤ *m.* *md _l_* ( *G*). Let *W* be the local *m*-resolving set of *G.* *Amal*( *G*, *v*, *m*) has *m* copies of *G*, such that $S=\cup_{j=1}^{j=m}W_{j}$ . We take identified vertex *v* ∉ *W*, thus two adjacent vertices *u*_*k*, *r*_, *u*_*k*, *s*_ ∈ *V* ( *G*) have different representation. There are two conditions for *u*_*k*, *r*_, *u*_*k*, *s*_ ∈ *V* ( *G*) to terminal vertex.

1. For *u*_*k*, *r*_, *u*_*k*, *s*_ ∈ *V* ( *G*) and *d* ( *u*_*k*, *r*_, *v*) = *d* ( *u*_*k*, *s*_, *v*), then *d* ( *u*_*k*, *r*_, *w*) = *d* ( *u*_*k*, *s*_, *w*) for *w* ∈ *S* − *W _k_.* Thus, *r _m_* ( *u*_*k*, *r*_| *S*)≠ *r _m_* ( *u*_*k*, *s*_| *S*).
2. For *u*_*k*, *r*_, *u*_*k*, *s*_ ∈ *V* ( *G*) and *d* ( *u*_*k*, *r*_, *v*)≠ *d* ( *u*_*k*, *s*_, *v*), then *d* ( *u*_*k*, *r*_, *w*)≠ *d* ( *u*_*k*, *s*_, *w*) for *w* ∈ *S* − *W _k_.* Thus, *r _m_* ( *u*_*k*, *r*_| *S*)≠ *r _m_* ( *u*_*k*, *s*_| *S*).

Based on 1 and 2 that $S=\cup_{j=1}^{j=m}W_{j}$ is the local *m*-resolving set of *Amal*( *G*, *v*, *m*). Thus, *md _l_* ( *Amal*( *G*, *v*, *m*)) ≤ *m.* *md _l_* ( *G*). □

Theorem 0.3
*If Amal* ( *P _n_*, *v*, *m*) *is an amalgamation of m paths, then md _l_* ( *Amal*( *P _n_*, *v*, *m*)) = 1

Proof.
Let

$$V\left(\text{Amal}\left(P_{n},v,m\right)\right)=\left\{u\right\}\cup \left\{u_{j,i};1\leq j\leq m,1\leq i\leq n-1\right\}$$

and

$$E\left(\text{Amal}\left(P_{n},v,m\right)\right)=\overset{j=m}{\underset{j=1}{\cup }}E\left({\left(P_{n}\right)}_{j}\right)\}$$

Since *P _n_* is bipartite graph, then *Amal* ( *P _n_*, *v*, *m*) is bipartite graph. Based on Theorem 0.1 that *md _l_* ( *Amal*( *P _n_*, *v*, *m*)) = 1. □

Theorem 0.4
*If Amal* ( *K _n_*, *v*, *m*) *is an amalgamation of m complete graphs, then* ${\mathrm{md}}_{l}\left(\text{Amal}\left(K_{n},v,m\right)\right)=\infty$

Proof.
Let

$$V\left(\text{Amal}\left(K_{n},v,m\right)\right)=\left\{u\right\}\cup \left\{u_{j,i};1\leq j\leq m,1\leq i\leq n-1\right\}$$

and

$$E\left(\text{Amal}\left(K_{n},v,m\right)\right)=\overset{j=m}{\underset{j=1}{\cup }}E\left({\left(K_{n}\right)}_{j}\right)\}$$

For two adjacent vertices *x*, *y* ∈ *V* (( *K _n_*) *_k_*) with *k* ∈ [1, *m*] such that we have *d*( *x*, *u*) = *d*( *y*, *u*). Since *d*( *x*, *u*) = *d*( *y*, *u*), then *d*( *x*, *w*) = *d*( *y*, *w*) with *w* ∈ *V* ( *Amal* ( *K _n_*, *v*, *m*)). Thus, *r _m_* ( *x*| *W*)≠ *r _m_* ( *y*| *W*). □

Consider graph *Amal* ( *W _n_*, *v*, *m*) where *v* is a center vertex of *W _n_.* Let *V* ( *Amal* ( *W _n_*, *v*, *m*)) = { *v*} ∪ { *u*_*j*, *i*_;1 ≤ *j* ≤ *m* and 1 ≤ *i* ≤ *n*} such that $E\left(\text{Amal}\left(W_{n},v,m\right)\right)=\cup_{j=1j=m}E({\left(W_{n}\right)}_{j})$ .

Theorem 0.5
*Let m and n be two integers with m* ≥ 2 *and n* ≥ 3. *Let W _n_ be a wheel graph of order n* + 1 *and v* ∈ *V* ( *W _n_*) *where v is a center vertex of W _n_*, *then md _l_* ( *Amal* ( *W _n_*, *v*, *m*)) = *m.* *md _l_* ( *W _n_*).

Proof.
Let *W* be a local *m*-resolvings of *W _n_* and *S* be a local *m*-resolving set of *Amal* ( *W _n_*, *v*, *m*) Based on Lemma 0.2 that *md _l_* ( *Amal*( *W _n_*, *v*, *m*)) ≤ *m.* *md _l_* ( *W _n_*). Furthermore, we will show that *md _l_* ( *Amal*( *W _n_*, *v*, *m*)) ≥ *m.* *md _l_* ( *W _n_*). Take any *S* ⊂ *V* ( *Amal* ( *W _n_*, *v*, *m*)) with | *P*|<| *S*|. Suppose | *P*|=| *S*| − 1 = *m.*| *W*| − 1. There is one copy of *W _n_*, ( *W _n_*) *_k_* have | *W _k_* |− 1, such that we have three condition as follows

i For *n* ≡ 0( *mod*4)
  Let *u*( *k*, *l*) ∉ *P* for 1 ≤ *l* ≤ *n* or *i* ≡ 4 *s* − 3 with $1\leq s\leq \frac{n}{4}$ and *d* ( *u*_*k*, 4( *s*−1)−3_, *u*_4( *s*+1)−3_) > 4, then $r_{m}\left(u_{k,4\left(s-1\right)-1}|W_{k}\right)=\ldots =r_{m}\left(u_{4\left(s+1\right)-5}|W_{k}\right)=\underset{\frac{n-4}{4}}{\underbrace{\left\{2,2,2,\ldots ,2\right\}}}$ . Since $d\left(u_{k,4\left(s-1\right)-1},w\right)=\ldots =d\left(u_{k,4\left(s+1\right)-5},w\right)$ for *w* ∈ *P* − *W _k_* and $r_{m}\left(u_{k,4\left(s-1\right)-1}|P\right)=\ldots =r_{m}\left(u_{k,4\left(s+1\right)-5}|P\right)$ , then *P* is not local m-resolving set of *Amal*( *W _n_*, *v*, *m*).
ii For *n* ≡ 2( *mod*4)
  There are three possibilities namely 1) *u*_*k*,1_ ∉ *P*; 2) *u*_*k*, *l*_ ∉ *P* for 1 < *l* < *n* − 1 and 3) *u*_*k*, *n*−1_ ∈ *P.*
  (a) Let *u*_*k*,1_ ∉ *P*, then $r_{m}\left(u_{k,1}|W_{k}\right)=\ldots =r_{m}\left(u_{k,3}|W_{k}\right)=\underset{\left\lceil \frac{n}{4}\right\rceil -1}{\underbrace{\left\{2,2,2,\ldots ,2\right\}}}$ . Since $d\left(u_{k,1},w\right)=\ldots =d\left(u_{k,3},w\right)$ for *w* ∈ *P* − *W _k_* and $r_{m}\left(u_{k,1}|P\right)=\ldots =r_{m}\left(u_{k,3}|P\right)$ , then *P* is not local m-resolving set of *Amal* ( *W _n_*, *v*, *m*).
  (b) Let *u*_*k*, *l*_ ∉ *P* for 1 < *l* < *n* − 1 or *i* ≡ 4 *s* − 3 with $2\leq k\leq \left\lceil \frac{n}{4}\right\rceil -1$ and *d* ( *u*_*k*, 4( *s*−1)−3_, *u*_*k*, 4( *s*+1)−3_) > 4, then $r_{m}\left(u_{k,4\left(s-1\right)-1}|W_{k}\right)=\ldots =r_{m}\left(u_{k,4\left(s+1\right)-5}|W_{k}\right)=\underset{\left\lceil \frac{n}{4}\right\rceil -1}{\underbrace{\left\{2,2,2,\ldots ,2\right\}}}$ . Since $d\left(u_{k,4\left(s-1\right)-1},w\right)=\ldots =d\left(u_{k,4\left(s+1\right)-5},w\right)$ for *w* ∈ *P* − *W _k_* and $r_{m}\left(u_{k,4\left(s-1\right)-1}|P\right)=\ldots =r_{m}\left(u_{k,4\left(s+1\right)-5}|P\right)$ , then *P* is not local m-resolving set of *Amal* ( *W _n_*, *v*, *m*).
  (c) Let *u*_*k*, *n*−1_ ∉ *P.* Since $r_{m}\left(u_{k,n-3}|W_{k}\right)=\ldots =r_{m}\left(u_{k,n-1}|W_{k}\right)=\underset{\left\lceil \frac{n}{4}\right\rceil -1}{\underbrace{\left\{2,2,2,\ldots ,2\right\}}}$ . Since $d\left(u_{k,n-3},w\right)=\ldots =d\left(u_{k,n-1},w\right)$ for *w* ∈ *P* − *W _k_* and $r_{m}\left(u_{k,n-3}|P\right)=\ldots =r_{m}\left(u_{k,n-1}|P\right)$ , then *P* is not local m-resolving set of *Amal* ( *W _n_*, *v*, *m*).
iii For *n* ≡ 1, 3( *mod*4)
  We know that ${\mathrm{md}}_{l}\left(W_{n}\right)=\infty$ , so there are two adjacent vertices *x*, *y* ∈ *V* ( *W _n_*), *r _m_* ( *x*| *W _k_*) = *r _m_* ( *y*| *W _k_*). Since *d*( *x*, *v*) = *d*( *y*, *v*) and *d*( *x*, *w*) = *d*( *x*, *w*) where *w* ∈ *P* − *W _k_*, then *r _m_* ( *x*| *P*) = *r _m_* ( *y*| *P*). Thus, ${\mathrm{md}}_{l}\left(\text{Amal}\left(W_{n},v,m\right)\right)=\infty$ .

Based on i), ii), and iii) that *md _l_* ( *Amal* ( *W _n_*, *v*, *m*)) = *m.* *md _l_* ( *W _n_*). □

For further discussion, consider graph *Amal* ( *W _n_*, *v*, *m*) where *v* is not a center vertex of *W _n_.* Let *V* ( *Amal* ( *W _n_*, *v*, *m*)) = { *v*} ∪ { *u _j_*;1 ≤ *j* ≤ *m*} ∪ { *u*_*j*, *i*_;1 ≤ *j* ≤ *m* and 1 ≤ *i* ≤ *n* − 1} such that *E* ( *Amal*( *W _n_*, *v*, *m*)) = { *vu _j_*, *vu*_*j*,1_, *vu*_*j*, *n*−1_}∪{ *u*_*j*, *i*_ *u*_*j*, *i*+1_;1 ≤ *i* ≤ *n*−2}∪{ *u _j_u*_*j*, *i*_;1 ≤ *j* ≤ *m*, 1 ≤ *i* ≤ *n*−1}.

Theorem 0.6
*Let m and n be two integers with m* ≥ 2 *and n* ≥ 3. *Let W _n_ be a wheel graph of order n* + 1 *and v* ∈ *V* ( *W _n_*) *where v is not center vertex of W _n_*, *then*

$${\mathrm{md}}_{l}\left(\text{Amal}\left(W_{n},v,m\right)\right)=\left\{\begin{array}{ll} m.\left(\frac{n}{4}-1\right), & \text{for}\;n\equiv 0\left(\mathrm{mod}\;4\right)\\ m.\lfloor \frac{n}{4}\rfloor , & \text{for}\;n\equiv 1,2,3\left(\mathrm{mod}4\right) \end{array}\right.$$

Proof.
We consider four cases.

Case 1.
For *n* ≡ 0(mod 4)

Choose *S* = { *u*_*j*, *i*_;1 ≤ *j* ≤ *m*, 1 ≤ *i* ≤ *n* − 1, *i* ≡ 0( *mod*4)} obtained the vertex representation as follows

1. For *u _k_*, *v* ∈ *V* ( *Amal* ( *W _n_*, *v*, *m*)), *d*( *v*, *w*)≠ *d* ( *u _k_*, *w*) where *w* ∈ *S* − *W _k_* such that *r _m_* ( *v*| *S* − *W _k_*)≠ *r _m_* ( *u _k_* | *S* − *W _k_*). Since *d*( *v*, *w*)≠ *d* ( *u _k_*, *w*) where *w* ∈ *W _k_* such that *r _m_* ( *v*| *W _k_*)≠ *r _m_* ( *u _k_* | *W _k_*). Thus, *r _m_* ( *v*| *S*)≠ *r _m_* ( *u _k_* | *S*).
2. For *u _k_*, *u*_*k*, *l*_ ∈ *V* (( *W _n_*) *_k_*) and *l*≠0( *mod*4). Since *u _k_* adjacent to *w* where *w* ∈ *W _k_*, such that *r _m_* ( *u _k_* | *W _k_*)≠ *r _m_* ( *u*_*k*, *l*_| *W _k_*). Since *d* ( *u _k_*, *w*)≠ *d* ( *u*_*k*, *l*_, *w*) where *w* ∈ *S* − *W _k_* such that *r _m_* ( *u _k_* | *S* − *W _k_*)≠ *r _m_* ( *u*_*k*, *l*_| *S* − *W _k_*) with *l*≠1, *n* − 1. Since *d* ( *u _k_*, *w*) = *d* ( *u*_*k*, *l*_, *w*) where *w* ∈ *S* − *W _k_* such that *r _m_* ( *u _k_* | *S* − *W _k_*) = *r _m_* ( *u*_*k*, *l*_| *S* − *W _k_*) with *l* = 1, *n* − 1. Thus, *r _m_* ( *v*| *S*)≠ *r _m_* ( *u _k_* | *S*).
3. For *u*_*k*, *i*_ ∈ *V* (( *W _n_*) *_k_*) and 1 ≤ *i* ≤ 3. Since *d* ( *u*_*k*,1_, *w*)≠ *d* ( *u*_*k*,2_, *w*) where *w* ∈ *S* − *W _k_* and *d* ( *u*_*k*,1_, *w*)≠ *d* ( *u*_*k*,2_, *w*) where *w* ∈ *W _k_* such that *r _m_* ( *u*_*k*,1_| *S*)≠ *r _m_* ( *u*_*k*,2_| *S*). Since *d* ( *u*_*k*,2_, *w*) = *d* ( *u*_*k*,3_, *w*) where *w* ∈ *S* − *W _k_* and *d* ( *u*_*k*,2_, *w*)≠ *d* ( *u*_*k*,3_, *w*) where *w* ∈ *W _k_* such that *r _m_* ( *u*_*k*,2_| *S*)≠ *r _m_* ( *u*_*k*,3_| *S*).
4. For *u*_*k*, *i*_ ∈ *V* (( *W _n_*) *_k_*) and *n* − 3 ≤ *i* ≤ *n* − 1. Since *d* ( *u*_*k*, *n*−1_, *w*)≠ *d* ( *u*_*k*, *n*−2_, *w*) where *w* ∈ *S* − *W _k_* and *d* ( *u*_*k*, *n*−1_, *w*)≠ *d* ( *u*_*k*, *n*−2_, *w*) where *w* ∈ *W _k_* such that *r _m_* ( *u*_*k*, *n*−1_| *S*)≠ *r _m_* ( *u*_*k*, *n*−2_| *S*). Since *d* ( *u*_*k*, *n*−2_, *w*) = *d* ( *u*_*k*, *n*−3_, *w*) where *w* ∈ *S* − *W _k_* and *d* ( *u*_*k*, *n*−2_, *w*)≠ *d* ( *u*_*k*, *n*−3_, *w*) where *w* ∈ *W _k_* such that *r _m_* ( *u*_*k*, *n*−2_| *S*)≠ *r _m_* ( *u*_*k*, *n*−3_| *S*).
5. For *u*_*k*, *i*_ ∈ *V* (( *W _n_*) *_k_*) where 4 *l* + 1 ≤ *i* ≤ 4 *l* + 3 and $1\leq l\leq \frac{n-8}{4}$ . Since *d* ( *u*_*k*,4 *l*+1_, *w*)≠ *d* ( *u*_*k*,4 *l*+2_, *w*) where *w* ∈ *S* − *W _k_* and *d* ( *u*_*k*,4 *l*+1_, *w*)≠ *d* ( *u*_*k*,4 *l*+2_, *w*) where *w* ∈ *W _k_* such that *r _m_* ( *u*_*k*,4 *l*+1_| *S*)≠ *r _m_* ( *u*_*k*,4 *l*+2_| *S*). Since *d* ( *u*_*k*,4 *l*+2_, *w*) = *d* ( *u*_*k*,4 *l*+3_, *w*) where *w* ∈ *S* − *W _k_* and *d* ( *u*_*k*,4 *l*+2_, *w*)≠ *d* ( *u*_*k*,4 *l*+3_, *w*) where *w* ∈ *W _k_* such that *r _m_* ( *u*_*k*,4 *l*+2_| *S*)≠ *r _m_* ( *u*_*k*, 4 *l*+3_| *S*).

Based on the representation above that every two adjacent vertices has distinct representations such that *S* is local m-resolving set and ${\mathrm{md}}_{l}\left(\text{Amal}\left(W_{n},v,m\right)\right)\leq m.\left(\frac{n}{4}-1\right)$ . Furthermore, proving that m ${\mathrm{md}}_{l}\left(\text{Amal}\left(W_{n},v,m\right)\right)\geq m.\left(\frac{n}{4}-1\right)$ . Taking any set *P* ⊂ *V* ( *Amal* ( *W _n_*, *v*, *m*)) with $|P|=m.\left(\frac{n}{4}-1\right)-1$ . Let *u*( *k*, *l*) ∉ *P* for *l* ≡ 4 *s* with $1\leq s\leq \frac{n-8}{4}$ and *d* ( *u*_*k*, 4( *s*−1)_, *u*_*k*,4( *s*+1)_) > 4, then $r_{m}\left(u_{k,4\left(s-1\right)+2}|W_{k}\right)=\ldots =r_{m}\left(u_{4\left(s+1\right)-2}|W_{k}\right)=\underset{\frac{n-8}{4}}{\underbrace{\left\{2,2,2,\ldots ,2\right\}}}$ . Since $d\left(u_{k,4\left(s-1\right)+2},w\right)=\ldots =d\left(u_{k,4\left(s+1\right)-2},w\right)$ for *w* ∈ *P* − *W _k_* and $r_{m}\left(u_{k,4\left(s-1\right)+2}|P\right)=\ldots =r_{m}\left(u_{k,4\left(s+1\right)-2}|P\right)$ , then *P* is not local m-resolving set of *Amal* ( *W _n_*, *v*, *m*). Thus, ${\mathrm{md}}_{l}\left(\text{Amal}\left(W_{n},v,m\right)\right)=m.\left(\frac{n}{4}-1\right)$ .

Case 2.
For *n* ≡ 1(mod 4)

Choose *S* = { *u*_*j*, *i*_;1 ≤ *j* ≤ *m*, 1 ≤ *i* ≤ *n* − 1, *i* ≡ 0( *mod*4)} obtained the vertex representation as follows

1. For *u _k_*, *v* ∈ *V* ( *Amal* ( *W _n_*, *v*, *m*)), *d*( *v*, *w*)≠ *d* ( *u _k_*, *w*) where *w* ∈ *S* − *W _k_* such that *r _m_* ( *v*| *S* − *W _k_*)≠ *r _m_* ( *u _k_* | *S* − *W _k_*). Since *d*( *v*, *w*)≠ *d* ( *u _k_*, *w*) where *w* ∈ *W _k_* such that *r _m_* ( *v*| *W _k_*)≠ *r _m_* ( *u _k_* | *W _k_*). Thus, *r _m_* ( *v*| *S*)≠ *r _m_* ( *u _k_* | *S*).
2. For *u _k_*, *u*_*k*, *l*_ ∈ *V* (( *W _n_*) *_k_*) and *l*≠0( *mod*4). Since *u _k_* adjacent to *w* where *w* ∈ *W _k_*, such that *r _m_* ( *u _k_* | *W _k_*)≠ *r _m_* ( *u*_*k*, *l*_| *W _k_*). Since *d* ( *u _k_*, *w*)≠ *d* ( *u*_*k*, *l*_, *w*) where *w* ∈ *S* − *W _k_* such that *r _m_* ( *u _k_* | *S* − *W _k_*)≠ *r _m_* ( *u*_*k*, *l*_| *S* − *W _k_*) with *l*≠1, *n* − 1. Since *d* ( *u _k_*, *w*) = *d* ( *u*_*k*, *l*_, *w*) where *w* ∈ *S* − *W _k_* such that *r _m_* ( *u _k_* | *S* − *W _k_*) = *r _m_* ( *u*_*k*, *l*_| *S* − *W _k_*) with *l* = 1, *n* − 1. Thus, *r _m_* ( *v*| *S*)≠ *r _m_* ( *u _k_* | *S*).
3. For *u*_*k*, *i*_ ∈ *V* (( *W _n_*) *_k_*) and 1 ≤ *i* ≤ 3. Since *d* ( *u*_*k*,1_, *w*)≠ *d* ( *u*_*k*,2_, *w*) where *w* ∈ *S* − *W _k_* and *d* ( *u*_*k*,1_, *w*)≠ *d* ( *u*_*k*,2_, *w*) where *w* ∈ *W _k_* such that *r _m_* ( *u*_*k*,1_| *S*)≠ *r _m_* ( *u*_*k*,2_| *S*). Since *d* ( *u*_*k*,2_, *w*) = *d* ( *u*_*k*,3_, *w*) where *w* ∈ *S* − *W _k_* and *d* ( *u*_*k*,2_, *w*)≠ *d* ( *u*_*k*,3_, *w*) where *w* ∈ *W _k_* such that *r _m_* ( *u*_*k*,2_| *S*)≠ *r _m_* ( *u*_*k*,3_| *S*).
4. For *u*_*k*, *i*_ ∈ *V* (( *W _n_*) *_k_*) where 4 *l* + 1 ≤ *i* ≤ 4 *l* + 3 and $1\leq l\leq \frac{n-5}{4}$ . Since *d* ( *u*_*k*,4 *l*+1_, *w*)≠ *d* ( *u*_*k*,4 *l*+2_, *w*) where *w* ∈ *S* − *W _k_* and *d* ( *u*_*k*,4 *l*+1_, *w*)≠ *d* ( *u*_*k*,4 *l*+2_, *w*) where *w* ∈ *W _k_* such that *r _m_* ( *u*_*k*,4 *l*+1_| *S*)≠ *r _m_* ( *u*_*k*,4 *l*+2_| *S*). Since *d* ( *u*_*k*,4 *l*+2_, *w*) = *d* ( *u*_*k*,4 *l*+3_, *w*) where *w* ∈ *S* − *W _k_* and *d* ( *u*_*k*,4 *l*+2_, *w*)≠ *d* ( *u*_*k*,4 *l*+3_, *w*) where *w* ∈ *W _k_* such that *r _m_* ( *u*_*k*,4 *l*+2_| *S*)≠ *r _m_* ( *u*_*k*,4 *l*+3_| *S*).

Based on the representation above that every two adjacent vertices has distinct representation such that *S* is local m-resolving set and ${\mathrm{md}}_{l}\left(\text{Amal}\left(W_{n},v,m\right)\right)\leq m.\left(\frac{n-1}{4}\right)$ . Furthermore, proving that m ${\mathrm{md}}_{l}\left(\text{Amal}\left(W_{n},v,m\right)\right)\geq m.\left(\frac{n-1}{4}\right)$ . Taking any set *P* ⊂ *V* ( *Amal* ( *W _n_*, *v*, *m*)) with $|P|=m.\left(\frac{n-1}{4}\right)-1$ . Let *u*_(_ *k*, *l*) ∉ *P* for *l* ≡ 4 *s* with $1\leq s\leq \frac{n-5}{4}$ and *d* ( *u*_*k*,4( *s*−1)_, *u*_*k*,4( *s*+1)_) > 4, then $r_{m}\left(u_{k,4\left(s-1\right)+2}|W_{k}\right)=\ldots =r_{m}\left(u_{4\left(s+1\right)-2}|W_{k}\right)=\underset{\frac{n-5}{4}}{\underbrace{\left\{2,2,2,\ldots ,2\right\}}}$ . Since $d\left(u_{k,4\left(s-1\right)+2},w\right)=\ldots =d\left(u_{k,4\left(s+1\right)-2},w\right)$ for *w* ∈ *P* − *W _k_* and $r_{m}\left(u_{k,4\left(s-1\right)+2}|P\right)=\ldots =r_{m}\left(u_{k,4\left(s+1\right)-2}|P\right)$ , then *P* is not local m-resolving set of *Amal* ( *W _n_*, *v*, *m*). Thus, ${\mathrm{md}}_{l}\left(\text{Amal}\left(W_{n},v,m\right)\right)=m.\left(\frac{n-1}{4}\right)$

Case 3.
For *n* ≡ 2(mod 4)

Choose *S* = { *u*_*j*, *i*_;1 ≤ *j* ≤ *m*, 1 ≤ *i* ≤ *n* − 1, *i* ≡ 0( *mod*4)} obtained the vertex representation as follows

1. For *u _k_*, *v* ∈ *V* ( *Amal* ( *W _n_*, *v*, *m*)), *d*( *v*, *w*)≠ *d* ( *u _k_*, *w*) where *w* ∈ *S* − *W _k_* such that *r _m_* ( *v*| *S* − *W _k_*)≠ *r _m_* ( *u _k_* | *S* − *W _k_*). Since *d*( *v*, *w*)≠ *d* ( *u _k_*, *w*) where *w* ∈ *W _k_* such that *r _m_* ( *v*| *W _k_*)≠ *r _m_* ( *u _k_* | *W _k_*). Thus, *r _m_* ( *v*| *S*)≠ *r _m_* ( *u _k_* | *S*).
2. For *u _k_*, *u*_*k*, *l*_ ∈ *V* (( *W _n_*) *_k_*) and *l*≠0( *mod*4). Since *u _k_* adjacent to *w* where *w* ∈ *W _k_*, such that *r _m_* ( *u _k_* | *W _k_*)≠ *r _m_* ( *u*_*k*, *l*_| *W _k_*). Since *d* ( *u _k_*, *w*)≠ *d* ( *u*_*k*, *l*_, *w*) where *w* ∈ *S* − *W _k_* such that *r _m_* ( *u _k_* | *S* − *W _k_*)≠ *r _m_* ( *u*_*k*, *l*_| *S* − *W _k_*) with *l*≠1, *n* − 1. Since *d* ( *u _k_*, *w*) = *d* ( *u*_*k*, *l*_, *w*) where *w* ∈ *S* − *W _k_* such that *r _m_* ( *u _k_* | *S* − *W _k_*) = *r _m_* ( *u*_*k*, *l*_| *S* − *W _k_*) with *l* = 1, *n* − 1. Thus, *r _m_* ( *v*| *S*)≠ *r _m_* ( *u _k_* | *S*).
3. For *u*_*k*, *i*_ ∈ *V* (( *W _n_*) *_k_*) and 1 ≤ *i* ≤ 3. Since *d* ( *u*_*k*,1_, *w*)≠ *d* ( *u*_*k*,2_, *w*) where *w* ∈ *S* − *W _k_* and *d* ( *u*_*k*,1_, *w*)≠ *d* ( *u*_*k*,2_, *w*) where *w* ∈ *W _k_* such that *r _m_* ( *u*_*k*,1_| *S*)≠ *r _m_* ( *u*_*k*,2_| *S*). Since *d* ( *u*_*k*,2_, *w*) = *d* ( *u*_*k*,3_, *w*) where *w* ∈ *S* − *W _k_* and *d* ( *u*_*k*,2_, *w*)≠ *d* ( *u*_*k*,3_, *w*) where *w* ∈ *W _k_* such that *r _m_* ( *u*_*k*,2_| *S*)≠ *r _m_* ( *u*_*k*,3_| *S*).
4. For *u*_*k*, *i*_ ∈ *V* (( *W _n_*) *_k_*) where 4 *l* + 1 ≤ *i* ≤ 4 *l* + 3 and $1\leq l\leq \frac{n-6}{4}$ . Since *d* ( *u*_*k*,4 *l*+1_, *w*)≠ *d* ( *u*_*k*,4 *l*+2_, *w*) where *w* ∈ *S* − *W _k_* and *d* ( *u*_*k*,4 *l*+1_, *w*)≠ *d* ( *u*_*k*,4 *l*+2_, *w*) where *w* ∈ *W _k_* such that *r _m_* ( *u*_*k*,4 *l*+1_| *S*)≠ *r _m_* ( *u*_*k*,4 *l*+2_| *S*). Since *d* ( *u*_*k*,4 *l*+2_, *w*) = *d* ( *u*_*k*,4 *l*+3_, *w*) where *w* ∈ *S* − *W _k_* and *d* ( *u*_*k*,4 *l*+2_, *w*)≠ *d* ( *u*_*k*,4 *l*+3_, *w*) where *w* ∈ *W _k_* such that *r _m_* ( *u*_*k*,4 *l*+2_| *S*)≠ *r _m_* ( *u*_*k*,4 *l*+3_| *S*).

Based on the representation above that every two adjacent vertices has distinct representation such that *S* is local m-resolving set and ${\mathrm{md}}_{l}\left(\text{Amal}\left(W_{n},v,m\right)\right)\leq m.\left(\frac{n-2}{4}\right)$ . Furthermore, proving that m ${\mathrm{md}}_{l}\left(\text{Amal}\left(W_{n},v,m\right)\right)\geq m.\left(\frac{n-2}{4}\right)$ . Taking any set *P* ⊂ *V* ( *Amal* ( *W _n_*, *v*, *m*)) with $|P|=m.\left(\frac{n-2}{4}\right)-1$ . Let *u*_(_ *k*, *l*) ∉ *P* for *l* ≡ 4 *s* with $1\leq s\leq \frac{n-6}{4}$ and *d* ( *u*_*k*,4( *s*−1)_, *u*_*k*,4( *s*+1)_) > 4, then $r_{m}\left(u_{k,4\left(s-1\right)+2}|W_{k}\right)=\ldots =r_{m}\left(u_{4\left(s+1\right)-2}|W_{k}\right)=\underset{\frac{n-6}{4}}{\underbrace{\left\{2,2,2,\ldots ,2\right\}}}$ . Since $d\left(u_{k,4\left(s-1\right)+2},w\right)=\ldots =d\left(u_{k,4\left(s+1\right)-2},w\right)$ for *w* ∈ *P* − *W _k_* and $r_{m}\left(u_{k,4\left(s-1\right)+2}|P\right)=\ldots =r_{m}\left(u_{k,4\left(s+1\right)-2}|P\right)$ , then *P* is not local m-resolving set of *Amal* ( *W _n_*, *v*, *m*). Thus, ${\mathrm{md}}_{l}\left(\text{Amal}\left(W_{n},v,m\right)\right)=m.\left(\frac{n-2}{4}\right)$

Case 4.
For *n* ≡ 3(mod 4)

Choose *S* = { *u*_*j*, *i*_;1 ≤ *j* ≤ *m*, 1 ≤ *i* ≤ *n* − 1, *i* ≡ 0( *mod*4)} obtained the vertex representation as follows

1. For *u _k_*, *v* ∈ *V* ( *Amal* ( *W _n_*, *v*, *m*)), *d*( *v*, *w*)≠ *d* ( *u _k_*, *w*) where *w* ∈ *S* − *W _k_* such that *r _m_* ( *v*| *S* − *W _k_*)≠ *r _m_* ( *u _k_* | *S* − *W _k_*). Since *d*( *v*, *w*)≠ *d* ( *u _k_*, *w*) where *w* ∈ *W _k_* such that *r _m_* ( *v*| *W _k_*)≠ *r _m_* ( *u _k_* | *W _k_*). Thus, *r _m_* ( *v*| *S*)≠ *r _m_* ( *u _k_* | *S*).
2. For *u _k_*, *u*_*k*, *l*_ ∈ *V* (( *W _n_*) *_k_*) and *l*≠0( *mod*4). Since *u _k_* adjacent to *w* where *w* ∈ *W _k_*, such that *r _m_* ( *u _k_* | *W _k_*)≠ *r _m_* ( *u*_*k*, *l*_| *W _k_*). Since *d* ( *u _k_*, *w*)≠ *d* ( *u*_*k*, *l*_, *w*) where *w* ∈ *S* − *W _k_* such that *r _m_* ( *u _k_* | *S* − *W _k_*)≠ *r _m_* ( *u*_*k*, *l*_| *S* − *W _k_*) with *l*≠1, *n* − 1. Since *d* ( *u _k_*, *w*) = *d* ( *u*_*k*, *l*_, *w*) where *w* ∈ *S* − *W _k_* such that *r _m_* ( *u _k_* | *S* − *W _k_*) = *r _m_* ( *u*_*k*, *l*_| *S* − *W _k_*) with *l* = 1, *n* − 1. Thus, *r _m_* ( *v*| *S*)≠ *r _m_* ( *u _k_* | *S*).
3. For *u*_*k*, *i*_ ∈ *V* (( *W _n_*) *_k_*) and 1 ≤ *i* ≤ 3. Since *d* ( *u*_*k*,1_, *w*)≠ *d* ( *u*_*k*,2_, *w*) where *w* ∈ *S* − *W _k_* and *d* ( *u*_*k*,1_, *w*)≠ *d* ( *u*_*k*,2_, *w*) where *w* ∈ *W _k_* such that *r _m_* ( *u*_*k*,1_| *S*)≠ *r _m_* ( *u*_*k*,2_| *S*). Since *d* ( *u*_*k*,2_, *w*) = *d* ( *u*_*k*,3_, *w*) where *w* ∈ *S* − *W _k_* and *d* ( *u*_*k*,2_, *w*)≠ *d* ( *u*_*k*,3_, *w*) where *w* ∈ *W _k_* such that *r _m_* ( *u*_*k*,2_| *S*)≠ *r _m_* ( *u*_*k*,3_| *S*).
4. For *u*_*k*, *i*_ ∈ *V* (( *W _n_*) *_k_*) and *n* − 2 ≤ *i* ≤ *n* − 1. Since *d* ( *u*_*k*, *n*−1_, *w*)≠ *d* ( *u*_*k*, *n*−2_, *w*) where *w* ∈ *S* − *W _k_* and *d* ( *u*_*k*, *n*−1_, *w*)≠ *d* ( *u*_*k*, *n*−2_, *w*) where *w* ∈ *W _k_* such that *r _m_* ( *u*_*k*, *n*−1_| *S*)≠ *r _m_* ( *u*_*k*, *n*−2_| *S*).
5. For *u*_*k*, *i*_ ∈ *V* (( *W _n_*) *_k_*) where 4 *l* + 1 ≤ *i* ≤ 4 *l* + 3 and $1\leq l\leq \frac{n-7}{4}$ . Since *d* ( *u*_*k*,4 *l*+1_, *w*)≠ *d* ( *u*_*k*,4 *l*+2_, *w*) where *w* ∈ *S* − *W _k_* and *d* ( *u*_*k*,4 *l*+1_, *w*)≠ *d* ( *u*_*k*,4 *l*+2_, *w*) where *w* ∈ *W _k_* such that *r _m_* ( *u*_*k*,4 *l*+1_| *S*)≠ *r _m_* ( *u*_*k*,4 *l*+2_| *S*). Since *d* ( *u*_*k*,4 *l*+2_, *w*) = *d* ( *u*_*k*,4 *l*+3_, *w*) where *w* ∈ *S* − *W _k_* and *d* ( *u*_*k*,4 *l*+2_, *w*)≠ *d* ( *u*_*k*,4 *l*+3_, *w*) where *w* ∈ *W _k_* such that *r _m_* ( *u*_*k*,4 *l*+2_| *S*)≠ *r _m_* ( *u*_*k*,4 *l*+3_| *S*).

Based on the representation above that every two adjacent vertices has distinct representations such that *S* is local m-resolving set and ${\mathrm{md}}_{l}\left(\text{Amal}\left(W_{n},v,m\right)\right)\leq m.\left(\frac{n-3}{4}\right)$ . Furthermore, proving that m ${\mathrm{md}}_{l}\left(\text{Amal}\left(W_{n},v,m\right)\right)\geq m.\left(\frac{n-3}{4}\right)$ . Taking any set *P* ⊂ *V* ( *Amal* ( *W _n_*, *v*, *m*)) with $|P|=m.\left(\frac{n-3}{4}\right)-1$ . Let *u*_(_ *k*, *l*) ∉ *P* for *l* ≡ 4 *s* with $1\leq s\leq \frac{n-7}{4}$ and *d* ( *u*_*k*, 4( *s*−1)_, *u*_*k*, 4( *s*+1)_) > 4, then $r_{m}\left(u_{k,4\left(s-1\right)+2}|W_{k}\right)=\ldots =r_{m}\left(u_{4\left(s+1\right)-2}|W_{k}\right)=\underset{\frac{n-7}{4}}{\underbrace{\left\{2,2,2,\ldots ,2\right\}}}$ . Since $d\left(u_{k,4\left(s-1\right)+2},w\right)=\ldots =d\left(u_{k,4\left(s+1\right)-2},w\right)$ for *w* ∈ *P* − *W _k_* and $r_{m}\left(u_{k,4\left(s-1\right)+2}|P\right)=\ldots =r_{m}\left(u_{k,4\left(s+1\right)-2}|P\right)$ , then *P* is not local m-resolving set of *Amal* ( *W _n_*, *v*, *m*). Thus, ${\mathrm{md}}_{l}\left(\text{Amal}\left(W_{n},v,m\right)\right)=m.\left(\frac{n-3}{4}\right)$ . □

Consider graph *Amal* ( *F _n_*, *v*, *m*) where *v* is a center vertex of *F _n_.* Let *V* ( *Amal* ( *F _n_*, *v*, *m*)) = { *v*} ∪ { *u*_*j*, *i*_;1 ≤ *j* ≤ *mand*1 ≤ *i* ≤ *n*} such that $E\left(\text{Amal}\left(F_{n},v,m\right)\right)=\cup_{j=1\;j=m}E\left({\left(F_{n}\right)}_{j}\right)$ .

Theorem 0.7
*Let m and n be two integers with m* ≥ 2 *and n* ≥ 6. *Let F _n_ be a fan graph of order n* + 1 *and v* ∈ *V* ( *F _n_*) *where v is a center vertex of F _n_*, *then md _l_* ( *Amal* ( *F _n_*, *v*, *m*)) = *m.* *md _l_* ( *F _n_*)

Proof.
Let *W* be a local *m*-resolvings of *F _n_* and *S* be a local *m*-resolving set of *Amal* ( *F _n_*, *v*, *m*) Based on Lemma 0.2 that *md _l_* ( *Amal*( *F _n_*, *v*, *m*)) ≤ *m.* *md _l_* ( *F _n_*). Furthermore, we will show that *md _l_* ( *Amal*( *F _n_*, *v*, *m*)) ≥ *m.* *md _l_* ( *F _n_*). Take any *S* ⊂ *V* ( *Amal* ( *F _n_*, *v*, *m*)) with | *P*|<| *S*|. Suppose | *P*|=| *S*| − 1 = *m.*| *W*| − 1. There are one copies of *F _n_*, ( *F _n_*) *_k_* have | *W _k_* |− 1, such that we have three condition as follows

i For *n* ≡ 1( *mod*4)
  There are three possibilities namely 1) *u*_*k*,3_ ∉ *P*; 2) *u*_*k*, *l*_ ∉ *P* for 3 < *l* < *n* − 2 and 3) *u*_*k*, *n*−2_ ∈ *P.*
  (a) Let *u*_*k*,3_ ∉ *P.* Since *u*_*k*,1_ *u*_*k*,2_ ∈ *E*(( *F _n_*) *_k_*), then $r_{m}\left(u_{k,1}|W_{k}\right)=r_{m}\left(u_{k,2}|W_{k}\right)=\underset{\frac{n-5}{4}}{\underbrace{\left\{2,2,2,\ldots ,2\right\}}}$ . Since *d* ( *u*_*k*,1_, *w*) = *d* ( *u*_*k*,2_, *w*) for *w* ∈ *P* − *W _k_* and *r _m_* ( *u*_*k*,1_| *P*) = *r _m_* ( *u*_*k*,2_| *P*), then *P* is not local m-resolving set of *Amal* ( *F _n_*, *v*, *m*).
  (b) Let *u*_*k*, *l*_ ∉ *P* for 3 < *l* < *n* − 2 or *i* ≡ 4 *s* − 1 with $2\leq s\leq \frac{n-5}{4}$ and *d* ( *u*_*k*, 4( *s*−1)−1_, *u*_*k*, 4( *s*+1)−1_) > 4, then $r_{m}\left(u_{k,4\left(s-1\right)+1}|W_{k}\right)=\ldots =r_{m}\left(u_{k,4\left(s+1\right)-3}|W_{k}\right)=\underset{\frac{n-5}{4}}{\underbrace{\left\{2,2,2,\ldots ,2\right\}}}$ . Since $d\left(u_{k,4\left(s-1\right)+1},w\right)=\ldots =d\left(u_{k,4\left(s+1\right)-3},w\right)$ for *w* ∈ *P* − *W _k_* and $r_{m}\left(u_{k,4\left(s-1\right)+1}|P\right)=\ldots =r_{m}\left(u_{k,4\left(s+1\right)-3}|P\right)$ , then *P* is not local m-resolving set of *Amal* ( *F _n_*, *v*, *m*).
  (c) Let *u*_*k*, *n*−2_ ∉ *P.* Since *u*_*k*, *n*−1_ *u*_*k*, *n*_ ∈ *E*(( *F _n_*) *_k_*), then $r_{m}\left(u_{k,n-4}|W_{k}\right)=\ldots =r_{m}\left(u_{k,n}|W_{k}\right)=\underset{\frac{n-5}{4}}{\underbrace{\left\{2,2,2,\ldots ,2\right\}}}$ . Since $d\left(u_{k,n-4},w\right)=\ldots =d\left(u_{k,n},w\right)$ for *w* ∈ *P* − *W _k_* and $r_{m}\left(u_{k,n-4}|P\right)=\ldots =r_{m}\left(u_{k,n}|P\right)$ , then *P* is not local m-resolving set of *Amal* ( *F _n_*, *v*, *m*).
ii For *n* ≡ 0, 2, 3( *mod*4)
  There are three possibilities namely 1) *u*_*k*,1_ ∉ *P*; 2) *u*_*k*, *l*_ ∉ *P* for 1 < *l* < *n* − 1 and 3) *u*_*k*, *n*−1_ ∈ *P* for *n* ≡ 0( *mod*4), *u*_*k*, *n*−1_ ∈ *P* for *n* ≡ 2( *mod*4), and *u*_*k*, *n*_ ∈ *P* for *n* ≡ 3( *mod*4).
  (a) Let *u*_*k*,3_ ∉ *P.* Since $r_{m}\left(u_{k,1}|W_{k}\right)=\ldots =r_{m}\left(u_{k,5}|W_{k}\right)=\underset{\left\lceil \frac{n}{4}\right\rceil -1}{\underbrace{\left\{2,2,2,\ldots ,2\right\}}}$ . Since $d\left(u_{k,1},w\right)=\ldots =d\left(u_{k,5},w\right)$ for *w* ∈ *P* − *W _k_* and $r_{m}\left(u_{k,1}|P\right)=\ldots =r_{m}\left(u_{k,5}|P\right)$ , then *P* is not local m-resolving set of *Amal* ( *F _n_*, *v*, *m*).
  (b) Let *u*_*k*, *l*_ ∉ *P* for 3 < *l* < *n* − 2 or *i* ≡ 4 *s* − 1 with $2\leq s\leq \left\lceil \frac{n}{4}\right\rceil$ and *d* ( *u*_*k*,4( *s*−1)−1_, *u*_*k*, 4( *s*+1)−1_) > 4, then $r_{m}\left(u_{k,4\left(s-1\right)+1}|W_{k}\right)=\ldots =r_{m}\left(u_{k,4\left(s+1\right)-3}|W_{k}\right)=\underset{\left\lceil \frac{n}{4}\right\rceil -1}{\underbrace{\left\{2,2,2,\ldots ,2\right\}}}$ . Since $d\left(u_{k,4\left(s-1\right)+1},w\right)=\ldots =d\left(u_{k,4\left(s+1\right)-3},w\right)$ for *w* ∈ *P* − *W _k_* and $r_{m}\left(u_{k,4\left(s-1\right)+1}|P\right)=\ldots =r_{m}\left(u_{k,4\left(s+1\right)-3}|P\right)$ , then *P* is not local m-resolving set of *Amal* ( *F _n_*, *v*, *m*).
  (c) Let *u*_*k*, *n*−1_ ∉ *P* for *n* ≡ 0( *mod*4). Since $r_{m}\left(u_{k,n-3}|W_{k}\right)=\ldots =r_{m}\left(u_{k,n}|W_{k}\right)=\underset{\left\lceil \frac{n}{4}\right\rceil -1}{\underbrace{\left\{2,2,2,\ldots ,2\right\}}}$ . Since $d\left(u_{k,n-3},w\right)=\ldots =d\left(u_{k,n},w\right)$ for *w* ∈ *P* − *W _k_* and $r_{m}\left(u_{k,n-3}|P\right)=\ldots =r_{m}\left(u_{k,n}|P\right)$ , then *P* is not local m-resolving set of *Amal* ( *F _n_*, *v*, *m*).
  (d) Let *u*_*k*, *n*−1_ ∉ *P* for *n* ≡ 2( *mod*4). Since $r_{m}\left(u_{k,n-1}|W_{k}\right)=r_{m}\left(u_{k,n}|W_{k}\right)=\underset{\left\lceil \frac{n}{4}\right\rceil -1}{\underbrace{\left\{2,2,2,\ldots ,2\right\}}}$ . Since *d* ( *u*_*k*, *n*−1_, *w*) = *d* ( *u*_*k*, *n*_, *w*) for *w* ∈ *P* − *W _k_* and *r _m_* ( *u*_*k*, *n*−1_| *P*) = *r _m_* ( *u*_*k*, *n*_| *P*), then *P* is not local m-resolving set of *Amal* ( *F _n_*, *v*, *m*).
  (e) Let *u*_*k*, *n*_ ∉ *P* for *n* ≡ 3( *mod*4). Since $r_{m}\left(u_{k,n-2}|W_{k}\right)=\ldots =r_{m}\left(u_{k,n}|W_{k}\right)=\underset{\left\lceil \frac{n}{4}\right\rceil -1}{\underbrace{\left\{2,2,2,\ldots ,2\right\}}}$ . Since $d\left(u_{k,n-2},w\right)=\ldots =d\left(u_{k,n},w\right)$ for *w* ∈ *P* − *W _k_* and $r_{m}\left(u_{k,n-2}|P\right)=\ldots =r_{m}\left(u_{k,n}|P\right)$ , then *P* is not local m-resolving set of *Amal* ( *F _n_*, *v*, *m*).

Based on i) and ii) that *md _l_* ( *Amal* ( *F _n_*, *v*, *m*)) = *m.* *md _l_* ( *F _n_*). □

For further discussion, consider graph *Amal* ( *F _n_*, *v*, *m*) where *v* ∈ *V* ( *F _n_*) with *d*( *v*) = 2. Let *V* ( *Amal* ( *F _n_*, *v*, *m*)) = { *v*} ∪ { *u _j_*;1 ≤ *j* ≤ *m*} ∪ { *u*_*j*, *i*_;1 ≤ *j* ≤ *mand*1 ≤ *i* ≤ *n* − 1} such that *E* ( *Amal*( *F _n_*, *v*, *m*)) = { *vu _j_*, *vu*_*j*,1_}∪{ *u*_*j*, *i*_ *u*_*j*, *i*+1_;1 ≤ *i* ≤ *n*−2}∪{ *u _j_u*_*j*, *i*_;1 ≤ *j* ≤ *m*, 1 ≤ *i* ≤ *n*−1}.

Theorem 0.8
*Let m and n be two integers with m* ≥ 2 *and n* ≥ 3. *Let F _n_ be a fan graph of order n* + 1 *and v* ∈ *V* ( *F _n_*) *where d*( *v*) = 2, *then* ${\mathrm{md}}_{l}\left(\text{Amal}\left(F_{n},v,m\right)\right)=m.\left\lfloor \frac{n}{4}\right\rfloor$

Proof.
We have some condition for the set *S* as follows

a for *n* ≡ 1, 2, 3( *mod*4), *S* = { *u*_*j*, *i*_;1 ≤ *j* ≤ *m*, 1 ≤ *i* ≤ *n* − 1, *i* ≡ 0( *mod*4)}.
b for *n* ≡ 0( *mod*4), *S* = { *u*_*j*, *i*_;1 ≤ *j* ≤ *m*, 1 ≤ *i* ≤ *n* − 1, *i* ≡ 0( *mod*4)} ∪ { *u*_*j*, *n*−2_}.

We obtained the vertex representation as follows

1. For *u _k_*, *v* ∈ *V* ( *Amal* ( *F _n_*, *v*, *m*)), *d*( *v*, *w*)≠ *d* ( *u _k_*, *w*) where *w* ∈ *S* − *W _k_* such that *r _m_* ( *v*| *S* − *W _k_*)≠ *r _m_* ( *u _k_* | *S* − *W _k_*). Since *d*( *v*, *w*)≠ *d* ( *u _k_*, *w*) where *w* ∈ *W _k_* such that *r _m_* ( *v*| *W _k_*)≠ *r _m_* ( *u _k_* | *W _k_*). Thus, *r _m_* ( *v*| *S*)≠ *r _m_* ( *u _k_* | *S*).
2. For *u _k_*, *u*_*k*, *l*_ ∈ *V* (( *F _n_*) *_k_*) where *l*≠0( *mod*4) and *u*_*k*, *l*_ where *l*≠ *n* − 2 for *n* ≡ 0( *mod*4). Since *u _k_* is adjacent to *w* where *w* ∈ *W _k_*, such that *r _m_* ( *u _k_* | *W _k_*)≠ *r _m_* ( *u*_*k*, *l*_| *W _k_*). Since *d* ( *u _k_*, *w*)≠ *d* ( *u*_*k*, *l*_, *w*) where *w* ∈ *S* − *W _k_* such that *r _m_* ( *u _k_* | *S* − *W _k_*)≠ *r _m_* ( *u*_*k*, *l*_| *S* − *W _k_*) with *l*≠1. Since *d* ( *u _k_*, *w*) = *d* ( *u*_*k*, *l*_, *w*) where *w* ∈ *S* − *W _k_* such that *r _m_* ( *u _k_* | *S* − *W _k_*) = *r _m_* ( *u*_*k*, *l*_| *S* − *W _k_*) with *l* = 1. Thus, *r _m_* ( *v*| *S*)≠ *r _m_* ( *u _k_* | *S*).
3. For *u*_*k*, *i*_ ∈ *V* (( *F _n_*) *_k_*) and 1 ≤ *i* ≤ 3. Since *d* ( *u*_*k*,1_, *w*)≠ *d* ( *u*_*k*,2_, *w*) where *w* ∈ *S* − *W _k_* and *d* ( *u*_*k*,1_, *w*)≠ *d* ( *u*_*k*,2_, *w*) where *w* ∈ *W _k_* such that *r _m_* ( *u*_*k*,1_| *S*)≠ *r _m_* ( *u*_*k*,2_| *S*). Since *d* ( *u*_*k*,2_, *w*) = *d* ( *u*_*k*,3_, *w*) where *w* ∈ *S* − *W _k_* and *d* ( *u*_*k*,2_, *w*)≠ *d* ( *u*_*k*,3_, *w*) where *w* ∈ *W _k_* such that *r _m_* ( *u*_*k*,2_| *S*)≠ *r _m_* ( *u*_*k*,3_| *S*).
4. For *u*_*k*, *i*_ ∈ *V* (( *F _n_*) *_k_*) where 4 *l* + 1 ≤ *i* ≤ 4 *l* + 3 and $1\leq l\leq \left\lfloor \frac{n}{4}\right\rfloor -1$ . Since *d* ( *u*_*k*,4 *l*+1_, *w*)≠ *d* ( *u*_*k*,4 *l*+2_, *w*) where *w* ∈ *S* − *W _k_* and *d* ( *u*_*k*,4 *l*+1_, *w*)≠ *d* ( *u*_*k*,4 *l*+2_, *w*) where *w* ∈ *W _k_* such that *r _m_* ( *u*_*k*,4 *l*+1_| *S*)≠ *r _m_* ( *u*_*k*,4 *l*+2_| *S*). Since *d* ( *u*_*k*,4 *l*+2_, *w*) = *d* ( *u*_*k*,4 *l*+3_, *w*) where *w* ∈ *S* − *W _k_* and *d* ( *u*_*k*,4 *l*+2_, *w*)≠ *d* ( *u*_*k*,4 *l*+3_, *w*) where *w* ∈ *W _k_* such that *r _m_* ( *u*_*k*,4 *l*+2_| *S*)≠ *r _m_* ( *u*_*k*,4 *l*+3_| *S*).
5. For *u*_*k*, *i*_ ∈ *V* (( *F _n_*) *_k_*) where *n* − 2 ≤ *i* ≤ *n* − 1 and *n* ≡ 3( *mod*4). Since *d* ( *u*_*k*, *n*−1_, *w*) = *d* ( *u*_*k*, *n*−2_, *w*) where *w* ∈ *S* − *W _k_* and *d* ( *u*_*k*, *n*−1_, *w*)≠ *d* ( *u*_*k*, *n*−2_, *w*) where *w* ∈ *W _k_* such that *r _m_* ( *u*_*k*, *n*−1_| *S*)≠ *r _m_* ( *u*_*k*, *n*−2_| *S*).

Based on the representation above that every two adjacent vertices has distinct representations such that *S* is local m-resolving set and ${\mathrm{md}}_{l}\left(\text{Amal}\left(W_{n},v,m\right)\right)\leq m.\left(\frac{n}{4}-1\right)$ .

Furthermore, we will show that ${\mathrm{md}}_{l}\left(\text{Amal}\left(F_{n},v,m\right)\right)\geq m.\left\lfloor \frac{n}{4}\right\rfloor$ . Take any *S* ⊂ *V* ( *Amal* ( *F _n_*, *v*, *m*)) with| *P*|<| *S*|. Suppose $|P|=|S|-1=m.\left\lfloor \frac{n}{4}\right\rfloor -1$ . There are one copies of *F _n_*, ( *F _n_*) *_k_* have $\left\lfloor \frac{n}{4}\right\rfloor -1$ , such that we have three condition as follows

i For *n* ≡ 1( *mod*4)
  There are three possibilities namely 1) *u*_*k*, *l*_ ∉ *P* for 1 < *l* < *n* − 1 and 2) *u*_*k*, *n*−1_ ∉ *P.*
  (a) Let *u*_*k*, *l*_ ∉ *P* for 1 < *l* ≤ *n* − 1 or *l* ≡ 4 *s* with $1\leq s\leq \frac{n-5}{4}$ and *d* ( *u*_*k*, 4( *s*−1)+1_, *u*_*k*, 4( *s*+1)−1_) > 4, then $r_{m}\left(u_{k,4\left(s-1\right)+2}|W_{k}\right)=\ldots =r_{m}\left(u_{k,4\left(s+1\right)-2}|W_{k}\right)=\underset{\frac{n-5}{4}}{\underbrace{\left\{2,2,2,\ldots ,2\right\}}}$ . Since $d\left(u_{k,4\left(s-1\right)+2},w\right)=\ldots =d\left(u_{k,4\left(s+1\right)-2},w\right)$ for *w* ∈ *P* − *W _k_* and $r_{m}\left(u_{k,4\left(s-1\right)+2}|P\right)=\ldots =r_{m}\left(u_{k,4\left(s+1\right)-2}|P\right)$ , then *P* is not local m-resolving set of *Amal* ( *F _n_*, *v*, *m*).
  (b) Let *u*_*k*, *n*−1_ ∉ *P.* Since $r_{m}\left(u_{k,n-3}|W_{k}\right)=\ldots =r_{m}\left(u_{k,n-1}|W_{k}\right)=\underset{\frac{n-5}{4}}{\underbrace{\left\{2,2,2,\ldots ,2\right\}}}$ . Since $d\left(u_{k,n-3},w\right)=\ldots =d\left(u_{k,n-1},w\right)$ for *w* ∈ *P* − *W _k_* and $r_{m}\left(u_{k,n-3}|P\right)=\ldots =r_{m}\left(u_{k,n-1}|P\right)$ , then *P* is not local m-resolving set of *Amal* ( *F _n_*, *v*, *m*).
ii For *n* ≡ 0, 2, 3( *mod*4)
  There are three possibilities namely 1) *u*_*k*, *l*_ ∉ *P* for 1 < *l* < *n* − 1 and 2) *u*_*k*, *n*−1_ ∈ *P* for *n* ≡ 0( *mod*4), *u*_*k*, *n*−2_ ∈ *P* for *n* ≡ 2( *mod*4), and *u*_*k*, *n*−3_ ∈ *P* for *n* ≡ 3( *mod*4).
  (a) Let *u*_*k*, *l*_ ∉ *P* for 1 < *l* < *n* − 1 or *l* ≡ 4 *s* with $1\leq s\leq \left\lfloor \frac{n}{4}\right\rfloor -1$ and *d* ( *u*_*k*, 4( *s*−1)+1_, *u*_*k*, 4( *s*+1)−1_) > 4, then $r_{m}\left(u_{k,4\left(s-1\right)+2}|W_{k}\right)=\ldots =r_{m}\left(u_{k,4\left(s+1\right)-2}|W_{k}\right)=\underset{\left\lfloor \frac{n}{4}\right\rfloor -1}{\underbrace{\left\{2,2,2,\ldots ,2\right\}}}$ . Since $d\left(u_{k,4\left(s-1\right)+2},w\right)=\ldots =d\left(u_{k,4\left(s+1\right)-2},w\right)$ for *w* ∈ *P* − *W _k_* and $r_{m}\left(u_{k,4\left(s-1\right)+2}|P\right)=\ldots =r_{m}\left(u_{k,4\left(s+1\right)-2}|P\right)$ , then *P* is not local m-resolving set of *Amal* ( *F _n_*, *v*, *m*).
  (b) Let *u*_*k*, *n*−1_ ∉ *P* for *n* ≡ 0( *mod*4). Since $r_{m}\left(u_{k,n-2}|W_{k}\right)=r_{m}\left(u_{k,n-1}|W_{k}\right)=\underset{\left\lfloor \frac{n}{4}\right\rfloor -1}{\underbrace{\left\{2,2,2,\ldots ,2\right\}}}$ . Since *d* ( *u*_*k*, *n*−2_, *w*) = *d* ( *u*_*k*, *n*−1_, *w*) for *w* ∈ *P* − *W _k_* and *r _m_* ( *u*_*k*, *n*−2_| *P*) = *r _m_* ( *u*_*k*, *n*−1_| *P*), then *P* is not local m-resolving set of *Amal* ( *F _n_*, *v*, *m*).
  (c) Let *u*_*k*, *n*−2_ ∉ *P* for *n* ≡ 2( *mod*4). Since $r_{m}\left(u_{k,n-4}|W_{k}\right)=\ldots =r_{m}\left(u_{k,n-1}|W_{k}\right)=\underset{\left\lfloor \frac{n}{4}\right\rfloor -1}{\underbrace{\left\{2,2,2,\ldots ,2\right\}}}$ . Since $d\left(u_{k,n-4},w\right)=\ldots =d\left(u_{k,n-1},w\right)$ for *w* ∈ *P* − *W _k_* and $r_{m}\left(u_{k,n-4}|P\right)=\ldots =r_{m}\left(u_{k,n-1}|P\right)$ , then *P* is not local m-resolving set of *Amal* ( *F _n_*, *v*, *m*).
  (d) Let *u*_*k*, *n*−3_ ∉ *P* for *n* ≡ 3( *mod*4). Since $r_{m}\left(u_{k,n-5}|W_{k}\right)=\ldots =r_{m}\left(u_{k,n-1}|W_{k}\right)=\underset{\left\lceil \frac{n}{4}\right\rceil -1}{\underbrace{\left\{2,2,2,\ldots ,2\right\}}}$ . Since $d\left(u_{k,n-5},w\right)=\ldots =d\left(u_{k,n-1},w\right)$ for *w* ∈ *P* − *W _k_* and $r_{m}\left(u_{k,n-5}|P\right)=\ldots =r_{m}\left(u_{k,n-1}|P\right)$ , then *P* is not local m-resolving set of *Amal* ( *F _n_*, *v*, *m*).

Based on i) and ii) that ${\mathrm{md}}_{l}\left(\text{Amal}\left(F_{n},v,m\right)\right)=m.\left\lfloor \frac{n}{4}\right\rfloor$ . □

Theorem 0.9
*Let m and n be two integers with m* ≥ 2 *and n* ≥ 3. *Let F _n_ be a fan graph of order n* + 1 *and v* ∈ *V* ( *F _n_*) *where d*( *v*) = 3, *then*

$${\mathrm{md}}_{l}\left(\text{Amal}\left(F_{n},v,m\right)\right)=\left\{\begin{array}{ll} m.\left(\left\lfloor \frac{p}{4}\right\rfloor +\left\lfloor \frac{n-p}{4}\right\rfloor \right), & \text{for}\;n-p\equiv 0,1,2\left(\mathrm{mod}4\right)\;\text{and}\;2\leq p\leq \left\lceil \frac{n}{4}\right\rceil \\ m.\left(\left\lfloor \frac{p}{4}\right\rfloor +\left\lceil \frac{n-p}{4}\right\rceil \right), & \text{for}\;n-p\equiv 3\left(\mathrm{mod}4\right)\;\text{and}\;2\leq p\leq \left\lceil \frac{n}{4}\right\rceil \end{array}\right.$$

Proof.
Since Identified vertex *v* = *u*_*j*, *p*_, then we have two part namely *u*_*j*,1_ − *u*_*j*, *p*−1_ and *u*_*j*, *p*+1_ − *u*_*j*, *n*_. We consider two cases as follows

Case 1.
For 2 ≤ *p* ≤ 3

For *d* ( *u*_*j*,1_, *u*_*j*, *p*−1_) ≤ 2, then the vertices $u_{j,1},\ldots ,u_{j,p-1}$ don’t need to be resolver. Thus, we focus for *d* ( *u*_*j*, *p*+1_, *u*_*j*, *n*_) ≥ 3

Sub Case 1.1.
For *n* − *p* ≡ 0, 1, 2( *mod*4)

Choose *S* = { *u*_*j*, *r*_;1 ≤ *j* ≤ *m*, *r* ≡ *p* ( *mod*4), *r* > *p*} obtained the vertex representation as follows

1. For *u*_*k*, *l*_ ∈ *V* ( *Amal* ( *F _n_*, *v*, *m*)) and 1 ≤ *l* ≤ *p* − 1, *d* ( *u*_*k*,1_, *w*)≠ *d* ( *u*_*k*,2_, *w*) where *w* ∈ *S* − *W _k_* such that *r _m_* ( *u*_*k*,1_| *S* − *W _k_*)≠ *r _m_* ( *u*_*k*,2_| *S* − *W _k_*). Since *d* ( *u*_*k*,1_, *w*) = *d* ( *u*_*k*,2_, *w*) where *w* ∈ *W _k_* such that *r _m_* ( *u*_*k*,1_| *W _k_*)≠ *r _m_* ( *u*_*k*,2_| *W _k_*). Thus, *r _m_* ( *u*_*k*,1_| *S*)≠ *r _m_* ( *u*_*k*,2_| *S*).
2. For *u _k_*, *v* ∈ *V* ( *Amal* ( *F _n_*, *v*, *m*)), *d*( *v*, *w*)≠ *d* ( *u _k_*, *w*) where *w* ∈ *S* − *W _k_* such that *r _m_* ( *v*| *S* − *W _k_*)≠ *r _m_* ( *u _k_* | *S* − *W _k_*). Since *d*( *v*, *w*)≠ *d* ( *u _k_*, *w*) where *w* ∈ *W _k_* such that *r _m_* ( *v*| *W _k_*)≠ *r _m_* ( *u _k_* | *W _k_*). Thus, *r _m_* ( *v*| *S*)≠ *r _m_* ( *u _k_* | *S*).
3. For *u _k_*, *u*_*k*, *l*_ ∈ *V* (( *F _n_*) *_k_*) where *l*≠ *p* ( *mod*4) and *u*_*k*, *l*_ where *l*≠ *n* − 2 for *n* − *p* ≡ 2( *mod*4). Since *u _k_* adjacent to *w* where *w* ∈ *W _k_*, such that *r _m_* ( *u _k_* | *W _k_*)≠ *r _m_* ( *u*_*k*, *l*_| *W _k_*). Since *d* ( *u _k_*, *w*)≠ *d* ( *u*_*k*, *l*_, *w*) where *w* ∈ *S* − *W _k_* such that *r _m_* ( *u _k_* | *S* − *W _k_*)≠ *r _m_* ( *u*_*k*, *l*_| *S* − *W _k_*) with *l*≠1. Since *d* ( *u _k_*, *w*) = *d* ( *u*_*k*, *l*_, *w*) where *w* ∈ *S* − *W _k_* such that *r _m_* ( *u _k_* | *S* − *W _k_*) = *r _m_* ( *u*_*k*, *l*_| *S* − *W _k_*) with *l* = 1. Thus, *r _m_* ( *v*| *S*)≠ *r _m_* ( *u _k_* | *S*).
4. For *u*_*k*, *i*_ ∈ *V* (( *F _n_*) *_k_*) and *p* + 1 ≤ *i* ≤ *p* + 3. Since *d* ( *u*_*k*, *p*+1_, *w*)≠ *d* ( *u*_*k*, *p*+2_, *w*) where *w* ∈ *S* − *W _k_* and *d* ( *u*_*k*, *p*+1_, *w*)≠ *d* ( *u*_*k*, *p*+2_, *w*) where *w* ∈ *W _k_* such that *r _m_* ( *u*_*k*, *p*+1_| *S*)≠ *r _m_* ( *u*_*k*, *p*+2_| *S*). Since *d* ( *u*_*k*, *p*+2_, *w*) = *d* ( *u*_*k*, *p*+3_, *w*) where *w* ∈ *S* − *W _k_* and *d* ( *u*_*k*, *p*+2_, *w*)≠ *d* ( *u*_*k*, *p*+3_, *w*) where *w* ∈ *W _k_* such that *r _m_* ( *u*_*k*, *p*+2_| *S*)≠ *r _m_* ( *u*_*k*, *p*+3_| *S*).
5. For *u*_*k*, *i*_ ∈ *V* (( *F _n_*) *_k_*) where *p* + 4 *l* + 1 ≤ *i* ≤ *p* + 4 *l* + 3 and $1\leq l\leq \left\lfloor \frac{n}{4}\right\rfloor -1$ . Since *d* ( *u*_*k*, *p*+4 *l*+1_, *w*)≠ *d* ( *u*_*k*, *p*+4 *l*+2_, *w*) where *w* ∈ *S* − *W _k_* and *d* ( *u*_*k*, *p*+4 *l*+1_, *w*)≠ *d* ( *u*_*k*, *p*+4 *l*+2_, *w*) where *w* ∈ *W _k_* such that *r _m_* ( *u*_*k*, *p*+4 *l*+1_| *S*)≠ *r _m_* ( *u*_*k*, *p*+4 *l*+2_| *S*). Since *d* ( *u*_*k*, *p*+4 *l*+2_, *w*) = *d* ( *u*_*k*, *p*+4 *l*+3_, *w*) where *w* ∈ *S* − *W _k_* and *d* ( *u*_*k*, *p*+4 *l*+2_, *w*)≠ *d* ( *u*_*k*, *p*+4 *l*+3_, *w*) where *w* ∈ *W _k_* such that *r _m_* ( *u*_*k*, *p*+4 *l*+2_| *S*)≠ *r _m_* ( *u*_*k*, *p*+4 *l*+3_| *S*).

Based on the representation above that every two adjacent vertices have distinct representations such that *S* is local m-resolving set and ${\mathrm{md}}_{l}\left(\text{Amal}\left(F_{n},v,m\right)\right)\leq m.\left(\left\lfloor \frac{p}{4}\right\rfloor +\left\lfloor \frac{n-p}{4}\right\rfloor \right)$ .

Furthermore, we will show that ${\mathrm{md}}_{l}\left(\text{Amal}\left(F_{n},v,m\right)\right)\geq m.\left(\left\lfloor \frac{p}{4}\right\rfloor +\left\lfloor \frac{n-p}{4}\right\rfloor \right)$ . Take any *S* ⊂ *V* ( *Amal* ( *F _n_*, *v*, *m*)) with | *P*|<| *S*|. Suppose $|P|=|S|-1=m.\left(\left\lfloor \frac{p}{4}\right\rfloor +\left\lfloor \frac{n-p}{4}\right\rfloor \right)-1$ . There is one copy of *F _n_*, ( *F _n_*) *_k_* have $m.\left(\left\lfloor \frac{p}{4}\right\rfloor +\left\lfloor \frac{n-p}{4}\right\rfloor \right)-1$ . Let *u*_*k*, *l*_ ∉ *P* for *p* + 1 < *l* ≤ *n* − 1 or *l* ≡ *p* + 4 *s* with $1\leq s\leq \left\lfloor \frac{n-p}{4}\right\rfloor$ and *d* ( *u*_*k*, *p*+4( *s*−1)+1_, *u*_*k*, *p*+4( *s*+1)−1_) > 4, then $r_{m}\left(u_{k,p+4\left(s-1\right)+2}|W_{k}\right)=\ldots =r_{m}\left(u_{k,p+4\left(s+1\right)-2}|W_{k}\right)=\underset{\left\lfloor \frac{n-p}{4}\right\rfloor -1}{\underbrace{\left\{2,2,2,\ldots ,2\right\}}}$ . Since $d\left(u_{k,p+4\left(s-1\right)+2},w\right)=\ldots =d\left(u_{k,p+4\left(s+1\right)-2},w\right)$ for *w* ∈ *P* − *W _k_* and $r_{m}\left(u_{k,p+4\left(s-1\right)+2}|P\right)=\ldots =r_{m}\left(u_{k,p+4\left(s+1\right)-2}|P\right)$ , then *P* is not local m-resolving set of *Amal* ( *F _n_*, *v*, *m*). Thus, ${\mathrm{md}}_{l}\left(\text{Amal}\left(F_{n},v,m\right)\right)=m.\left(\left\lfloor \frac{p}{4}\right\rfloor +\left\lfloor \frac{n-p}{4}\right\rfloor \right)$ .

Sub Case 1.2.
For *n* − *p* ≡ 3( *mod*4)

Choose *S* = { *u*_*j*, *r*_;1 ≤ *j* ≤ *m*, *r* ≡ *p* ( *mod*4), *r* > *p*} ∪ { *u*_*j*, *n*−1_} obtained the vertex representation as follows

1. For *u*_*k*, *l*_ ∈ *V* ( *Amal* ( *F _n_*, *v*, *m*)) and 1 ≤ *l* ≤ *p* − 1, *d* ( *u*_*k*,1_, *w*)≠ *d* ( *u*_*k*,2_, *w*) where *w* ∈ *S* − *W _k_* such that *r _m_* ( *u*_*k*,1_| *S* − *W _k_*)≠ *r _m_* ( *u*_*k*,2_| *S* − *W _k_*). Since *d* ( *u*_*k*,1_, *w*) = *d* ( *u*_*k*,2_, *w*) where *w* ∈ *W _k_* such that *r _m_* ( *u*_*k*,1_| *W _k_*)≠ *r _m_* ( *u*_*k*,2_| *W _k_*). Thus, *r _m_* ( *u*_*k*,1_| *S*)≠ *r _m_* ( *u*_*k*,2_| *S*).
2. For *u _k_*, *v* ∈ *V* ( *Amal* ( *F _n_*, *v*, *m*)), *d*( *v*, *w*)≠ *d* ( *u _k_*, *w*) where *w* ∈ *S* − *W _k_* such that *r _m_* ( *v*| *S* − *W _k_*)≠ *r _m_* ( *u _k_* | *S* − *W _k_*). Since *d*( *v*, *w*)≠ *d* ( *u _k_*, *w*) where *w* ∈ *W _k_* such that *r _m_* ( *v*| *W _k_*)≠ *r _m_* ( *u _k_* | *W _k_*). Thus, *r _m_* ( *v*| *S*)≠ *r _m_* ( *u _k_* | *S*).
3. For *u _k_*, *u*_*k*, *l*_ ∈ *V* (( *F _n_*) *_k_*) where *l*≠ *p* ( *mod*4) and *u*_*k*, *l*_ where *l*≠ *n* − 2 for *n* − *p* ≡ 3( *mod*4). Since *u _k_* adjacent to *w* where *w* ∈ *W _k_*, such that *r _m_* ( *u _k_* | *W _k_*)≠ *r _m_* ( *u*_*k*, *l*_| *W _k_*). Since *d* ( *u _k_*, *w*)≠ *d* ( *u*_*k*, *l*_, *w*) where *w* ∈ *S* − *W _k_* such that *r _m_* ( *u _k_* | *S* − *W _k_*)≠ *r _m_* ( *u*_*k*, *l*_| *S* − *W _k_*) with *l*≠1. Since *d* ( *u _k_*, *w*) = *d* ( *u*_*k*, *l*_, *w*) where *w* ∈ *S* − *W _k_* such that *r _m_* ( *u _k_* | *S* − *W _k_*) = *r _m_* ( *u*_*k*, *l*_| *S* − *W _k_*) with *l* = 1. Thus, *r _m_* ( *v*| *S*)≠ *r _m_* ( *u _k_* | *S*).
4. For *u*_*k*, *i*_ ∈ *V* (( *F _n_*) *_k_*) and *p* + 1 ≤ *i* ≤ *p* + 3. Since *d* ( *u*_*k*, *p*+1_, *w*)≠ *d* ( *u*_*k*, *p*+2_, *w*) where *w* ∈ *S* − *W _k_* and *d* ( *u*_*k*, *p*+1_, *w*)≠ *d* ( *u*_*k*, *p*+2_, *w*) where *w* ∈ *W _k_* such that *r _m_* ( *u*_*k*, *p*+1_| *S*)≠ *r _m_* ( *u*_*k*, *p*+2_| *S*). Since *d* ( *u*_*k*, *p*+2_, *w*) = *d* ( *u*_*k*, *p*+3_, *w*) where *w* ∈ *S* − *W _k_* and *d* ( *u*_*k*, *p*+2_, *w*)≠ *d* ( *u*_*k*, *p*+3_, *w*) where *w* ∈ *W _k_* such that *r _m_* ( *u*_*k*, *p*+2_| *S*)≠ *r _m_* ( *u*_*k*, *p*+3_| *S*).
5. For *u*_*k*, *i*_ ∈ *V* (( *F _n_*) *_k_*) where *p* + 4 *l* + 1 ≤ *i* ≤ *p* + 4 *l* + 3 and $1\leq l\leq \left\lfloor \frac{n}{4}\right\rfloor -1$ . Since *d* ( *u*_*k*, *p*+4 *l*+1_, *w*)≠ *d* ( *u*_*k*, *p*+4 *l*+2_, *w*) where *w* ∈ *S* − *W _k_* and *d* ( *u*_*k*, *p*+4 *l*+1_, *w*)≠ *d* ( *u*_*k*, *p*+4 *l*+2_, *w*) where *w* ∈ *W _k_* such that *r _m_* ( *u*_*k*, *p*+4 *l*+1_| *S*)≠ *r _m_* ( *u*_*k*, *p*+4 *l*+2_| *S*). Since *d* ( *u*_*k*, *p*+4 *l*+2_, *w*) = *d* ( *u*_*k*, *p*+4 *l*+3_, *w*) where *w* ∈ *S* − *W _k_* and *d* ( *u*_*k*, *p*+4 *l*+2_, *w*)≠ *d* ( *u*_*k*, *p*+4 *l*+3_, *w*) where *w* ∈ *W _k_* such that *r _m_* ( *u*_*k*, *p*+4 *l*+2_| *S*)≠ *r _m_* ( *u*_*k*, *p*+4 *l*+3_| *S*).
6. For *u*_*k*, *i*_ ∈ *V* (( *F _n_*) *_k_*) where *i* = *n* − 2, *n.* Since *u*_*k*, *n*−2_ is not adjacent to *u*_*k*, *n*_. It is clear.

Based on the representation above that every two adjacent vertices has distinct representations such that *S* is local m-resolving set and ${\mathrm{md}}_{l}\left(\text{Amal}\left(F_{n},v,m\right)\right)\leq m.\left(\left\lfloor \frac{p}{4}\right\rfloor +\left\lceil \frac{n-p}{4}\right\rceil \right)$ .

Furthermore, we will show that ${\mathrm{md}}_{l}\left(\text{Amal}\left(F_{n},v,m\right)\right)\geq m.\left(\left\lfloor \frac{p}{4}\right\rfloor +\left\lceil \frac{n-p}{4}\right\rceil \right)$ . Take any *S* ⊂ *V* ( *Amal* ( *F _n_*, *v*, *m*)) with| *P*|<| *S*|. Suppose $|P|=|S|-1=m.\left(\left\lfloor \frac{p}{4}\right\rfloor +\left\lceil \frac{n-p}{4}\right\rceil \right)-1$ . There are one copies of *F _n_*, ( *F _n_*) *_k_* have $m.\left(\left\lfloor \frac{p}{4}\right\rfloor +\left\lceil \frac{n-p}{4}\right\rceil \right)-1$ . There are three possibilities namely 1) *u*_*k*, *l*_ ∉ *P* for 1 < *l* < *n* − 1 and 2) *u*_*k*, *n*−1_ ∉ *P.*

1. Let *u*_*k*, *l*_ ∉ *P* for 1 < *l* ≤ *n* − 1 or *l* ≡ *p* + 4 *s* with $1\leq s\leq \left\lfloor \frac{n-p}{4}\right\rfloor$ and *d* ( *u*_*k*, *p*+4( *s*−1)+1_, *u*_*k*, *p*+4( *s*+1)−1_) > 4, then $r_{m}\left(u_{k,p+4\left(s-1\right)+2}|W_{k}\right)=\ldots =r_{m}\left(u_{k,p+4\left(s+1\right)-2}|W_{k}\right)=\underset{\left\lfloor \frac{n-p}{4}\right\rfloor }{\underbrace{\left\{2,2,2,\ldots ,2\right\}}}$ . Since $d\left(u_{k,p+4\left(s-1\right)+2},w\right)=\ldots =d\left(u_{k,p+4\left(s+1\right)-2},w\right)$ for *w* ∈ *P* − *W _k_* and $r_{m}\left(u_{k,p+4\left(s-1\right)+2}|P\right)=\ldots =r_{m}\left(u_{k,p+4\left(s+1\right)-2}|P\right)$ , then *P* is not local m-resolving set of *Amal* ( *F _n_*, *v*, *m*).
2. Let *u*_*k*, *n*−1_ ∉ *P.* Since $r_{m}\left(u_{k,n-1}|W_{k}\right)=r_{m}\left(u_{k,n}|W_{k}\right)=\underset{\left\lfloor \frac{n-p}{4}\right\rfloor }{\underbrace{\left\{2,2,2,\ldots ,2\right\}}}$ . Since *d* ( *u*_*k*, *n*−1_, *w*) = *d* ( *u*_*k*, *n*_, *w*) for *w* ∈ *P* − *W _k_* and *r _m_* ( *u*_*k*, *n*−1_| *P*) = *r _m_* ( *u*_*k*, *n*_| *P*), then *P* is not local m-resolving set of *Amal* ( *F _n_*, *v*, *m*).

Based on 1) and 2) that ${\mathrm{md}}_{l}\left(\text{Amal}\left(F_{n},v,m\right)\right)\geq m.\left(\left\lfloor \frac{p}{4}\right\rfloor +\left\lceil \frac{n-p}{4}\right\rceil \right)$ . Thus, ${\mathrm{md}}_{l}\left(\text{Amal}\left(F_{n},v,m\right)\right)=m.\left(\left\lfloor \frac{p}{4}\right\rfloor +\left\lceil \frac{n-p}{4}\right\rceil \right)$ .

Case 2.
For $4\leq p\leq \left\lceil \frac{n}{4}\right\rceil$

Sub Case 2.1.
For *n* − *p* ≡ 0, 1, 2( *mod*4)

We have some conditions for the set *S* as follows:

a for *p* ≡ 1( *mod*4), *S* = { *u*_*j*, *t*_;1 ≤ *j* ≤ *m*, *t* ≡ 1( *mod*4)} ∪ { *u*_*j*, *r*_;1 ≤ *j* ≤ *m*, *r* ≡ *p* ( *mod*4), *r* > *p*}.
b for *p* ≡ 2( *mod*4), *S* = { *u*_*j*, *t*_;1 ≤ *j* ≤ *m*, *t* ≡ 2( *mod*4)} ∪ { *u*_*j*, *r*_;1 ≤ *j* ≤ *m*, *r* ≡ *p* ( *mod*4), *r* > *p*}.
c for *p* ≡ 3( *mod*4), *S* = { *u*_*j*, *t*_;1 ≤ *j* ≤ *m*, *t* ≡ 3( *mod*4)} ∪ { *u*_*j*, *r*_;1 ≤ *j* ≤ *m*, *r* ≡ *p* ( *mod*4), *r* > *p*}.
d for *p* ≡ 0( *mod*4), *S* = { *u*_*j*, *t*_;1 ≤ *j* ≤ *m*, *t* ≡ 0( *mod*4)} ∪ { *u*_*j*, *r*_;1 ≤ *j* ≤ *m*, *r* ≡ *p* ( *mod*4), *r* > *p*}.

We obtained the vertex representation as follows:

1. For *u*_*k*, *i*_ ∈ *V* (( *F _n_*) *_k_*) where *p* − 3 ≤ *i* ≤ *p* − 1 and *p* ≡ 0, 1, 2, 3( *mod*4). Since *d* ( *u*_*k*, *p*−1_, *w*)≠ *d* ( *u*_*k*, *p*−2_, *w*) where *w* ∈ *S* − *W _k_* and *d* ( *u*_*k*, *p*−1_, *w*)≠ *d* ( *u*_*k*, *p*−2_, *w*) where *w* ∈ *W _k_* such that *r _m_* ( *u*_*k*, *p*−1_| *S*)≠ *r _m_* ( *u*_*k*, *p*−2_| *S*). Since *d* ( *u*_*k*, *p*−2_, *w*) = *d* ( *u*_*k*, *p*−3_, *w*) where *w* ∈ *S* − *W _k_* and *d* ( *u*_*k*, *p*−2_, *w*)≠ *d* ( *u*_*k*, *p*−3_, *w*) where *w* ∈ *W _k_* such that *r _m_* ( *u*_*k*, *p*−2_| *S*)≠ *r _m_* ( *u*_*k*, *p*−3_| *S*).
2. For *u*_*k*, *i*_ ∈ *V* (( *F _n_*) *_k_*) where *p* − 4 *l* + 1 ≤ *i* ≤ *p* − 4 *l* + 3, $2\leq l\leq \left\lfloor \frac{n}{4}\right\rfloor -1$ , and a *p* ≡ 0, 1, 2, 3( *mod*4). Since *d* ( *u*_*k*, *p*−4 *l*+1_, *w*)≠ *d* ( *u*_*k*, *p*−4 *l*+2_, *w*) where *w* ∈ *S* − *W _k_* and *d* ( *u*_*k*, *p*−4 *l*+1_, *w*)≠ *d* ( *u*_*k*, *p*−4 *l*+2_, *w*) where *w* ∈ *W _k_* such that *r _m_* ( *u*_*k*, *p*−4 *l*+1_| *S*)≠ *r _m_* ( *u*_*k*, *p*−4 *l*+2_| *S*). Since *d* ( *u*_*k*, *p*−4 *l*+2_, *w*) = *d* ( *u*_*k*, *p*−4 *l*+3_, *w*) where *w* ∈ *S* − *W _k_* and *d* ( *u*_*k*, *p*−4 *l*+2_, *w*)≠ *d* ( *u*_*k*, *p*−4 *l*+3_, *w*) where *w* ∈ *W _k_* such that *r _m_* ( *u*_*k*, *p*−4 *l*+2_| *S*)≠ *r _m_* ( *u*_*k*, *p*−4 *l*+3_| *S*).
3. For *u*_*k*, *l*_ ∈ *V* ( *Amal* ( *F _n_*, *v*, *m*)) and 1 ≤ *l* ≤ *p* − 1, *d* ( *u*_*k*,1_, *w*)≠ *d* ( *u*_*k*,2_, *w*) where *w* ∈ *S* − *W _k_* such that *r _m_* ( *u*_*k*,1_| *S* − *W _k_*)≠ *r _m_* ( *u*_*k*,2_| *S* − *W _k_*). Since *d* ( *u*_*k*,1_, *w*) = *d* ( *u*_*k*,2_, *w*) where *w* ∈ *W _k_* such that *r _m_* ( *u*_*k*,1_| *W _k_*)≠ *r _m_* ( *u*_*k*,2_| *W _k_*). Thus, *r _m_* ( *u*_*k*,1_| *S*)≠ *r _m_* ( *u*_*k*,2_| *S*).
4. For *u _k_*, *v* ∈ *V* ( *Amal* ( *F _n_*, *v*, *m*)), *d*( *v*, *w*)≠ *d* ( *u _k_*, *w*) where *w* ∈ *S* − *W _k_* such that *r _m_* ( *v*| *S* − *W _k_*)≠ *r _m_* ( *u _k_* | *S* − *W _k_*). Since *d*( *v*, *w*)≠ *d* ( *u _k_*, *w*) where *w* ∈ *W _k_* such that *r _m_* ( *v*| *W _k_*)≠ *r _m_* ( *u _k_* | *W _k_*). Thus, *r _m_* ( *v*| *S*)≠ *r _m_* ( *u _k_* | *S*).
5. For *u _k_*, *u*_*k*, *l*_ ∈ *V* (( *F _n_*) *_k_*) where *l*≠ *p* ( *mod*4) and *u*_*k*, *l*_ where *l*≠ *n* − 2 for *n* − *p* ≡ 2( *mod*4). Since *u _k_* adjacent to *w* where *w* ∈ *W _k_*, such that *r _m_* ( *u _k_* | *W _k_*)≠ *r _m_* ( *u*_*k*, *l*_| *W _k_*). Since *d* ( *u _k_*, *w*)≠ *d* ( *u*_*k*, *l*_, *w*) where *w* ∈ *S* − *W _k_* such that *r _m_* ( *u _k_* | *S* − *W _k_*)≠ *r _m_* ( *u*_*k*, *l*_| *S* − *W _k_*) with *l*≠1. Since *d* ( *u _k_*, *w*) = *d* ( *u*_*k*, *l*_, *w*) where *w* ∈ *S* − *W _k_* such that *r _m_* ( *u _k_* | *S* − *W _k_*) = *r _m_* ( *u*_*k*, *l*_| *S* − *W _k_*) with *l* = 1. Thus, *r _m_* ( *v*| *S*)≠ *r _m_* ( *u _k_* | *S*).
6. For *u*_*k*, *i*_ ∈ *V* (( *F _n_*) *_k_*) and *p* + 1 ≤ *i* ≤ *p* + 3. Since *d* ( *u*_*k*, *p*+1_, *w*)≠ *d* ( *u*_*k*, *p*+2_, *w*) where *w* ∈ *S* − *W _k_* and *d* ( *u*_*k*, *p*+1_, *w*)≠ *d* ( *u*_*k*, *p*+2_, *w*) where *w* ∈ *W _k_* such that *r _m_* ( *u*_*k*, *p*+1_| *S*)≠ *r _m_* ( *u*_*k*, *p*+2_| *S*). Since *d* ( *u*_*k*, *p*+2_, *w*) = *d* ( *u*_*k*, *p*+3_, *w*) where *w* ∈ *S* − *W _k_* and *d* ( *u*_*k*, *p*+2_, *w*)≠ *d* ( *u*_*k*, *p*+3_, *w*) where *w* ∈ *W _k_* such that *r _m_* ( *u*_*k*, *p*+2_| *S*)≠ *r _m_* ( *u*_*k*, *p*+3_| *S*).
7. For *u*_*k*, *i*_ ∈ *V* (( *F _n_*) *_k_*) where *p* + 4 *l* + 1 ≤ *i* ≤ *p* + 4 *l* + 3 and $1\leq l\leq \left\lfloor \frac{n}{4}\right\rfloor -1$ . Since *d* ( *u*_*k*, *p*+4 *l*+1_, *w*)≠ *d* ( *u*_*k*, *p*+4 *l*+2_, *w*) where *w* ∈ *S* − *W _k_* and *d* ( *u*_*k*, *p*+4 *l*+1_, *w*)≠ *d* ( *u*_*k*, *p*+4 *l*+2_, *w*) where *w* ∈ *W _k_* such that *r _m_* ( *u*_*k*, *p*+4 *l*+1_| *S*)≠ *r _m_* ( *u*_*k*, *p*+4 *l*+2_| *S*). Since *d* ( *u*_*k*, *p*+4 *l*+2_, *w*) = *d* ( *u*_*k*, *p*+4 *l*+3_, *w*) where *w* ∈ *S* − *W _k_* and *d* ( *u*_*k*, *p*+4 *l*+2_, *w*)≠ *d* ( *u*_*k*, *p*+4 *l*+3_, *w*) where *w* ∈ *W _k_* such that *r _m_* ( *u*_*k*, *p*+4 *l*+2_| *S*)≠ *r _m_* ( *u*_*k*, *p*+4 *l*+3_| *S*).

Based on the representation above that every two adjacent vertices has distinct representations such that *S* is a local m-resolving set and ${\mathrm{md}}_{l}\left(\text{Amal}\left(F_{n},v,m\right)\right)\leq m.\left(\left\lfloor \frac{p}{4}\right\rfloor +\left\lfloor \frac{n-p}{4}\right\rfloor \right)$ .

Furthermore, we will show that ${\mathrm{md}}_{l}\left(\text{Amal}\left(F_{n},v,m\right)\right)\geq m.\left(\left\lfloor \frac{p}{4}\right\rfloor +\left\lfloor \frac{n-p}{4}\right\rfloor \right)$ . Take any *S* ⊂ *V* ( *Amal* ( *F _n_*, *v*, *m*)) with| *P*|<| *S*|. Suppose $|P|=|S|-1=m.\left(\left\lfloor \frac{p}{4}\right\rfloor +\left\lfloor \frac{n-p}{4}\right\rfloor \right)-1$ . There are one copies of *F _n_*, ( *F _n_*) *_k_* have $m.\left(\left\lfloor \frac{p}{4}\right\rfloor +\left\lfloor \frac{n-p}{4}\right\rfloor \right)-1$ .

1. Let *u*_*k*, *l*_ ∉ *P* for *p* + 1 < *l* ≤ *n* − 1 or *l* ≡ *p* + 4 *s* with $1\leq s\leq \left\lfloor \frac{n-p}{4}\right\rfloor$ and *d* ( *u*_*k*, *p*+4( *s*−1)+1_, *u*_*k*, *p*+4( *s*+1)−1_) > 4, then $r_{m}\left(u_{k,p+4\left(s-1\right)+2}|W_{k}\right)=\ldots =r_{m}\left(u_{k,p+4\left(s+1\right)-2}|W_{k}\right)=\underset{\left\lfloor \frac{n-p}{4}\right\rfloor -1}{\underbrace{\left\{2,2,2,\ldots ,2\right\}}}$ . Since $d\left(u_{k,p+4\left(s-1\right)+2},w\right)=\ldots =d\left(u_{k,p+4\left(s+1\right)-2},w\right)$ for *w* ∈ *P* − *W _k_* and $r_{m}\left(u_{k,p+4\left(s-1\right)+2}|P\right)=\ldots =r_{m}\left(u_{k,p+4\left(s+1\right)-2}|P\right)$ , then *P* is not local m-resolving set of *Amal* ( *F _n_*, *v*, *m*).
2. Let *u*_*k*, *l*_ ∉ *P* for 1 < *l* ≤ *p* − 1 or *l* ≡ 1( *mod*4) and *p* ≡ 1( *mod*4), then $r_{m}\left(u_{k,p-4s-2}|W_{k}\right)=\ldots =r_{m}\left(u_{k,p-4s+2}|W_{k}\right)=\underset{\left\lfloor \frac{n-p}{4}\right\rfloor -1}{\underbrace{\left\{2,2,2,\ldots ,2\right\}}}$ . Since $d\left(u_{k,p-4s-2},w\right)=\ldots =d\left(u_{k,p-4s+2},w\right)$ for *w* ∈ *P* − *W _k_* and $r_{m}\left(u_{k,p-4s-2}|P\right)=\ldots =r_{m}\left(u_{k,p-4s+2}|P\right)$ , then *P* is not local m-resolving set of *Amal* ( *F _n_*, *v*, *m*).

Thus, ${\mathrm{md}}_{l}\left(\text{Amal}\left(F_{n},v,m\right)\right)=m.\left(\left\lfloor \frac{p}{4}\right\rfloor +\left\lfloor \frac{n-p}{4}\right\rfloor \right)$ .

Sub Case 2.2.
For *n* − *p* ≡ 3( *mod*4)

We have some conditions for the set *S* as follows

a for *p* ≡ 1( *mod*4), *S* = { *u*_*j*, *t*_;1 ≤ *j* ≤ *m*, *t* ≡ 1( *mod*4)}∪{ *u*_*j*, *r*_;1 ≤ *j* ≤ *m*, *r* ≡ *p* ( *mod*4), *r* > *p*}∪{ *u*_*j*, *n*−1_}.
b for *p* ≡ 2( *mod*4), *S* = { *u*_*j*, *t*_;1 ≤ *j* ≤ *m*, *t* ≡ 2( *mod*4)}∪{ *u*_*j*, *r*_;1 ≤ *j* ≤ *m*, *r* ≡ *p* ( *mod*4), *r* > *p*}∪{ *u*_*j*, *n*−1_}.
c for *p* ≡ 3( *mod*4), *S* = { *u*_*j*, *t*_;1 ≤ *j* ≤ *m*, *t* ≡ 3( *mod*4)}∪{ *u*_*j*, *r*_;1 ≤ *j* ≤ *m*, *r* ≡ *p* ( *mod*4), *r* > *p*}∪{ *u*_*j*, *n*−1_}.
d for *p* ≡ 0( *mod*4), *S* = { *u*_*j*, *t*_;1 ≤ *j* ≤ *m*, *t* ≡ 0( *mod*4)}∪{ *u*_*j*, *r*_;1 ≤ *j* ≤ *m*, *r* ≡ *p* ( *mod*4), *r* > *p*}∪{ *u*_*j*, *n*−1_}.

We obtained the vertex representation as follows:

1. For *u*_*k*, *i*_ ∈ *V* (( *F _n_*) *_k_*) where *p* − 3 ≤ *i* ≤ *p* − 1 and *p* ≡ 0, 1, 2, 3( *mod*4). Since *d* ( *u*_*k*, *p*−1_, *w*)≠ *d* ( *u*_*k*, *p*−2_, *w*) where *w* ∈ *S* − *W _k_* and *d* ( *u*_*k*, *p*−1_, *w*)≠ *d* ( *u*_*k*, *p*−2_, *w*) where *w* ∈ *W _k_* such that *r _m_* ( *u*_*k*, *p*−1_| *S*)≠ *r _m_* ( *u*_*k*, *p*−2_| *S*). Since *d* ( *u*_*k*, *p*−2_, *w*) = *d* ( *u*_*k*, *p*−3_, *w*) where *w* ∈ *S* − *W _k_* and *d* ( *u*_*k*, *p*−2_, *w*)≠ *d* ( *u*_*k*, *p*−3_, *w*) where *w* ∈ *W _k_* such that *r _m_* ( *u*_*k*, *p*−2_| *S*)≠ *r _m_* ( *u*_*k*, *p*−3_| *S*).
2. For *u*_*k*, *i*_ ∈ *V* (( *F _n_*) *_k_*) where *p* − 4 *l* + 1 ≤ *i* ≤ *p* − 4 *l* + 3, $2\leq l\leq \left\lfloor \frac{n}{4}\right\rfloor -1$ , and a *p* ≡ 0, 1, 2, 3( *mod*4). Since *d* ( *u*_*k*, *p*−4 *l*+1_, *w*)≠ *d* ( *u*_*k*, *p*−4 *l*+2_, *w*) where *w* ∈ *S* − *W _k_* and *d* ( *u*_*k*, *p*−4 *l*+1_, *w*)≠ *d* ( *u*_*k*, *p*−4 *l*+2_, *w*) where *w* ∈ *W _k_* such that *r _m_* ( *u*_*k*, *p*−4 *l*+1_| *S*)≠ *r _m_* ( *u*_*k*, *p*−4 *l*+2_| *S*). Since *d* ( *u*_*k*, *p*−4 *l*+2_, *w*) = *d* ( *u*_*k*, *p*−4 *l*+3_, *w*) where *w* ∈ *S* − *W _k_* and *d* ( *u*_*k*, *p*−4 *l*+2_, *w*)≠ *d* ( *u*_*k*, *p*−4 *l*+3_, *w*) where *w* ∈ *W _k_* such that *r _m_* ( *u*_*k*, *p*−4 *l*+2_| *S*)≠ *r _m_* ( *u*_*k*, *p*−4 *l*+3_| *S*).
3. For *u*_*k*, *l*_ ∈ *V* ( *Amal* ( *F _n_*, *v*, *m*)) and 1 ≤ *l* ≤ *p* − 1, *d* ( *u*_*k*,1_, *w*)≠ *d* ( *u*_*k*,2_, *w*) where *w* ∈ *S* − *W _k_* such that *r _m_* ( *u*_*k*,1_| *S* − *W _k_*)≠ *r _m_* ( *u*_*k*,2_| *S* − *W _k_*). Since *d* ( *u*_*k*,1_, *w*) = *d* ( *u*_*k*,2_, *w*) where *w* ∈ *W _k_* such that *r _m_* ( *u*_*k*,1_| *W _k_*)≠ *r _m_* ( *u*_*k*,2_| *W _k_*). Thus, *r _m_* ( *u*_*k*,1_| *S*)≠ *r _m_* ( *u*_*k*,2_| *S*).
4. For *u _k_*, *v* ∈ *V* ( *Amal* ( *F _n_*, *v*, *m*)), *d*( *v*, *w*)≠ *d* ( *u _k_*, *w*) where *w* ∈ *S* − *W _k_* such that *r _m_* ( *v*| *S* − *W _k_*)≠ *r _m_* ( *u _k_* | *S* − *W _k_*). Since *d*( *v*, *w*)≠ *d* ( *u _k_*, *w*) where *w* ∈ *W _k_* such that *r _m_* ( *v*| *W _k_*)≠ *r _m_* ( *u _k_* | *W _k_*). Thus, *r _m_* ( *v*| *S*)≠ *r _m_* ( *u _k_* | *S*).
5. For *u _k_*, *u*_*k*, *l*_ ∈ *V* (( *F _n_*) *_k_*) where *l*≠ *p* ( *mod*4) and *u*_*k*, *l*_ where *l*≠ *n* − 2 for *n* − *p* ≡ 3( *mod*4). Since *u _k_* adjacent to *w* where *w* ∈ *W _k_*, such that *r _m_* ( *u _k_* | *W _k_*)≠ *r _m_* ( *u*_*k*, *l*_| *W _k_*). Since *d* ( *u _k_*, *w*)≠ *d* ( *u*_*k*, *l*_, *w*) where *w* ∈ *S* − *W _k_* such that *r _m_* ( *u _k_* | *S* − *W _k_*)≠ *r _m_* ( *u*_*k*, *l*_| *S* − *W _k_*) with *l*≠1. Since *d* ( *u _k_*, *w*) = *d* ( *u*_*k*, *l*_, *w*) where *w* ∈ *S* − *W _k_* such that *r _m_* ( *u _k_* | *S* − *W _k_*) = *r _m_* ( *u*_*k*, *l*_| *S* − *W _k_*) with *l* = 1. Thus, *r _m_* ( *v*| *S*)≠ *r _m_* ( *u _k_* | *S*).
6. For *u*_*k*, *i*_ ∈ *V* (( *F _n_*) *_k_*) and *p* + 1 ≤ *i* ≤ *p* + 3. Since *d* ( *u*_*k*, *p*+1_, *w*)≠ *d* ( *u*_*k*, *p*+2_, *w*) where *w* ∈ *S* − *W _k_* and *d* ( *u*_*k*, *p*+1_, *w*)≠ *d* ( *u*_*k*, *p*+2_, *w*) where *w* ∈ *W _k_* such that *r _m_* ( *u*_*k*, *p*+1_| *S*)≠ *r _m_* ( *u*_*k*, *p*+2_| *S*). Since *d* ( *u*_*k*, *p*+2_, *w*) = *d* ( *u*_*k*, *p*+3_, *w*) where *w* ∈ *S* − *W _k_* and *d* ( *u*_*k*, *p*+2_, *w*)≠ *d* ( *u*_*k*, *p*+3_, *w*) where *w* ∈ *W _k_* such that *r _m_* ( *u*_*k*, *p*+2_| *S*)≠ *r _m_* ( *u*_*k*, *p*+3_| *S*).
7. For *u*_*k*, *i*_ ∈ *V* (( *F _n_*) *_k_*) where *p* + 4 *l* + 1 ≤ *i* ≤ *p* + 4 *l* + 3 and $1\leq l\leq \left\lfloor \frac{n}{4}\right\rfloor -1$ . Since *d* ( *u*_*k*, *p*+4 *l*+1_, *w*)≠ *d* ( *u*_*k*, *p*+4 *l*+2_, *w*) where *w* ∈ *S* − *W _k_* and *d* ( *u*_*k*, *p*+4 *l*+1_, *w*)≠ *d* ( *u*_*k*, *p*+4 *l*+2_, *w*) where *w* ∈ *W _k_* such that *r _m_* ( *u*_*k*, *p*+4 *l*+1_| *S*)≠ *r _m_* ( *u*_*k*, *p*+4 *l*+2_| *S*). Since *d* ( *u*_*k*, *p*+4 *l*+2_, *w*) = *d* ( *u*_*k*, *p*+4 *l*+3_, *w*) where *w* ∈ *S* − *W _k_* and *d* ( *u*_*k*, *p*+4 *l*+2_, *w*)≠ *d* ( *u*_*k*, *p*+4 *l*+3_, *w*) where *w* ∈ *W _k_* such that *r _m_* ( *u*_*k*, *p*+4 *l*+2_| *S*)≠ *r _m_* ( *u*_*k*, *p*+4 *l*+3_| *S*).
8. For *u*_*k*, *i*_ ∈ *V* (( *F _n_*) *_k_*) where *i* = *n* − 2, *n.* Since *u*_*k*, *n*−2_ does not adjacent to *u*_*k*, *n*_. It is clear.

Based on the representation above that every two adjacent vertices have distinct representations such that *S* is a local m-resolving set and ${\mathrm{md}}_{l}\left(\text{Amal}\left(F_{n},v,m\right)\right)\leq m.\left(\left\lfloor \frac{p}{4}\right\rfloor +\left\lceil \frac{n-p}{4}\right\rceil \right)$ .

Furthermore, we will show that ${\mathrm{md}}_{l}\left(\text{Amal}\left(F_{n},v,m\right)\right)\geq m.\left(\left\lfloor \frac{p}{4}\right\rfloor +\left\lceil \frac{n-p}{4}\right\rceil \right)$ . Take any *S* ⊂ *V* ( *Amal* ( *F _n_*, *v*, *m*)) with| *P*|<| *S*|. Suppose $|P|=|S|-1=m.\left(\left\lfloor \frac{p}{4}\right\rfloor +\left\lceil \frac{n-p}{4}\right\rceil \right)-1$ . There are one copies of *F _n_*, ( *F _n_*) *_k_* have $m.\left(\left\lfloor \frac{p}{4}\right\rfloor +\left\lceil \frac{n-p}{4}\right\rceil \right)-1$ . There are three possibilities namely 1) *u*_*k*, *l*_ ∉ *P* for 1 < *l* < *n* − 1 and 2) *u*_*k*, *n*−1_ ∉ *P.*

1. Let *u*_*k*, *l*_ ∉ *P* for 1 < *l* ≤ *n* − 1 or *l* ≡ *p* + 4 *s* with $1\leq s\leq \left\lfloor \frac{n-p}{4}\right\rfloor$ and *d* ( *u*_*k*, *p*+4( *s*−1)+1_, *u*_*k*, *p*+4( *s*+1)−1_) > 4, then $r_{m}\left(u_{k,p+4\left(s-1\right)+2}|W_{k}\right)=\ldots =r_{m}\left(u_{k,p+4\left(s+1\right)-2}|W_{k}\right)=\underset{\left\lfloor \frac{n-p}{4}\right\rfloor }{\underbrace{\left\{2,2,2,\ldots ,2\right\}}}$ . Since $d\left(u_{k,p+4\left(s-1\right)+2},w\right)=\ldots =d\left(u_{k,p+4\left(s+1\right)-2},w\right)$ for *w* ∈ *P* − *W _k_* and $r_{m}\left(u_{k,p+4\left(s-1\right)+2}|P\right)=\ldots =r_{m}\left(u_{k,p+4\left(s+1\right)-2}|P\right)$ , then *P* is not local m-resolving set of *Amal* ( *F _n_*, *v*, *m*).
2. Let *u*_*k*, *n*−1_ ∉ *P.* Since $r_{m}\left(u_{k,n-1}|W_{k}\right)=r_{m}\left(u_{k,n}|W_{k}\right)=\underset{\left\lfloor \frac{n-p}{4}\right\rfloor }{\underbrace{\left\{2,2,2,\ldots ,2\right\}}}$ . Since *d* ( *u*_*k*, *n*−1_, *w*) = *d* ( *u*_*k*, *n*_, *w*) for *w* ∈ *P* − *W _k_* and *r _m_* ( *u*_*k*, *n*−1_| *P*) = *r _m_* ( *u*_*k*, *n*_| *P*), then *P* is not local m-resolving set of *Amal* ( *F _n_*, *v*, *m*).
3. Let *u*_*k*, *l*_ ∉ *P* for 1 < *l* ≤ *p* − 1 or *l* ≡ 1( *mod*4) and *p* ≡ 1( *mod*4), then $r_{m}\left(u_{k,p-4s-2}|W_{k}\right)=\ldots =r_{m}\left(u_{k,p-4s+2}|W_{k}\right)=\underset{\left\lfloor \frac{n-p}{4}\right\rfloor -1}{\underbrace{\left\{2,2,2,\ldots ,2\right\}}}$ . Since $d\left(u_{k,p-4s-2},w\right)=\ldots =d\left(u_{k,p-4s+2},w\right)$ for *w* ∈ *P* − *W _k_* and $r_{m}\left(u_{k,p-4s-2}|P\right)=\ldots =r_{m}\left(u_{k,p-4s+2}|P\right)$ , then *P* is not local m-resolving set of *Amal* ( *F _n_*, *v*, *m*).

Based on 1) and 2) that ${\mathrm{md}}_{l}\left(\text{Amal}\left(F_{n},v,m\right)\right)\geq m.\left(\left\lfloor \frac{p}{4}\right\rfloor +\left\lceil \frac{n-p}{4}\right\rceil \right)$ . Thus, ${\mathrm{md}}_{l}\left(\text{Amal}\left(F_{n},v,m\right)\right)=m.\left(\left\lfloor \frac{p}{4}\right\rfloor +\left\lceil \frac{n-p}{4}\right\rceil \right)$ . □

## Conclusion

We have characterized the local multiset dimension of amalgamation graphs. We have found the upper bound of local multiset dimension and determined the exact value of local multiset dimension of path *P _n_*, complete graph *K _n_*, wheel graph *W _n_*, and fan graph *F _n_.* There are some graphs that attain the upper bound of local multiset dimension namely wheel graphs. On the otherhand, we found the following problem, as follows.

Open Problem 0.1 Determine the lower bound of local multiset dimension of amalgamation graphs.

## Data Availability

No data are associated with this article.
