# Supplementary material for: Local Multiset Dimension of Amalgamation Graphs
Source: F1000Res. 2024 Apr 23;12:95. Originally published 2023 Jan 24. [Version 2] doi: 10.12688/f1000research.128866.2 (PMC11148532; doi:10.12688/f1000research.128866.2)
Supplement: Peer review history

# Reviewer report: Reviewer response for version 2

**DOI:** 10.5256/f1000research.163253.r268983

**Published:** 2024-06-03

**Authors:** Muhammad Kamran Siddique

**Affiliations:** 1 Department of Mathematics, COMSATS University Islamabad, Lahore, Pakistan

The authors have carefully addressed all the reviewing comments raised in the first round of review. It's my pleasure to recommend acceptance of the current version.

Is the work clearly and accurately presented and does it cite the current literature?

Yes

If applicable, is the statistical analysis and its interpretation appropriate?

Yes

Are all the source data underlying the results available to ensure full reproducibility?

Partly

Is the study design appropriate and is the work technically sound?

Yes

Are the conclusions drawn adequately supported by the results?

Yes

Are sufficient details of methods and analysis provided to allow replication by others?

Yes

Reviewer Expertise:

Graph theory. Discrete Mathematics, Chemical graph Theory, Combinatorics

I confirm that I have read this submission and believe that I have an appropriate level of expertise to confirm that it is of an acceptable scientific standard.

# Reviewer report: Reviewer response for version 1

**DOI:** 10.5256/f1000research.141499.r161414

**Published:** 2023-10-31

**Authors:** Muhammad Kamran Siddique

**Affiliations:** 1 Department of Mathematics, COMSATS University Islamabad, Lahore, Pakistan

Local Multiset Dimension of Amalgamation Graphs

 I found that all the mathematical work is correct. Moreover paper is clearly written in a good way. It is new, as well as good contribution in the current area of research.

 Corrections:

1. The definitions of *m*-resolving set and local *m*-resolving set conveys the same meaning. The condition *xy=E(G)*, should be removed from the definition of *m*-resolving set.
2. On page no. 4, local multiset dimension is denoted by *μ*_*l*_ *(G)*. But *m* *d*_*l*_ *(G)* is used in definition and other results.
3. In the illustration of local *m*-resolving set, *W={* *v*_*1*_ *}* is the considered local *m*-resolving set, but the vertex representations are given with respect to Π.
4. In figure 2, the terminal vertex *v*_*0*_ is not labelled in the graphs.
5. In the line above Lemma 0.2, it is mentioned that upper bound for *Amal(G,v,m)* is given.
6. In the proof of Lemma 0.2, the terminal vertex *v* is not included in the vertex set of *Amal(G,v,m)*. Instead, a vertex *u* is mentioned. The same mistake can be seen in Theorem 0.3 and Theorem 0.4.
7. On page no. 6, in the proof of Lemma 0.2, it is given that *u*_*k,r*_ *_,_* *u*_*k,s*_ *∈V(G)* are at equal distance from every *w∈S-* *W*_*k*_ and it is concluded that their representations differ with respect to *W*.
8. In the edge set of *Amal(* *P*_*n*_ *,v,m)* [Theorem 0.3] and *Amal(* *K*_*n*_ *,v,m)* [Theorem 0.4],  an extra curly brace is placed.
9. In the proof of Theorem 0.4, two adjacent vertices *x* and *y* are mentioned to be equidistant from every *w∈V(Amal(* *K*_*n*_ *,v,m))*. Followed by that, it is given that they have different representation w.r.t *W*.
10. In the edge set of *Amal(* *W*_*n*_ *,v,m)*, *j=1* *m* *E(* *W* *n* *j* *)* is typed as *j=1 j=m* *E(* *W* *n* *j* *)*. Similarly, for *F*_*n*_
11.  The set *P i*s not mentioned clearly in proof of Theorem 0.5, Theorem 0.7, Theorem 0.8 and Theorem 0.9.
12. In the proof of Theorem 0.7, (page no. 10, first line) ‘local *m*-resolvings of *F*_*n*’_ should be changed as ‘local *m*-resolving set of *F*_*n*’._
13. It is mentioned in many places that there are three possibilities but followed by that only two cases are given.
14. Cite following recent paper about this topic ^1^^-^^6^.

Is the work clearly and accurately presented and does it cite the current literature?

Yes

If applicable, is the statistical analysis and its interpretation appropriate?

Yes

Are all the source data underlying the results available to ensure full reproducibility?

Partly

Is the study design appropriate and is the work technically sound?

Yes

Are the conclusions drawn adequately supported by the results?

Yes

Are sufficient details of methods and analysis provided to allow replication by others?

Yes

Reviewer Expertise:

Graph theory. Discrete Mathematics, Chemical graph Theory, Combinatorics

I confirm that I have read this submission and believe that I have an appropriate level of expertise to confirm that it is of an acceptable scientific standard, however I have significant reservations, as outlined above.

## References

[1] : Edge-Version Atom-Bond Connectivity and Geometric Arithmetic Indices of Generalized Bridge Molecular Graphs. *Symmetry* .2018;10(12) : 10.3390/sym10120751 10.3390/sym10120751

[2] : Multiplicative Zagreb Indices of Molecular Graphs. *Journal of Chemistry* .2019;2019: 10.1155/2019/5294198 1-19 10.1155/2019/5294198

[3] : On Degree Based Topological Properties of Two Carbon Nanotubes. *Polycyclic Aromatic Compounds* .2022;42(3) : 10.1080/10406638.2020.1753221 866-884 10.1080/10406638.2020.1753221

[4] : The Cartesian Product and Join Graphs on Edge-Version Atom-Bond Connectivity and Geometric Arithmetic Indices. *Molecules* .2018;23(7) : 10.3390/molecules23071731 10.3390/molecules23071731 30012953 PMC6100620

[5] : Study of Hardness of Superhard Crystals by Topological Indices. *Journal of Chemistry* .2021;2021: 10.1155/2021/9604106 1-10 10.1155/2021/9604106

[6] : Physical Analysis of Heat for Formation and Entropy of Ceria Oxide Using Topological Indices. *Comb Chem High Throughput Screen* .2022;25(3) : 10.2174/1386207323999201001210832 441-450 10.2174/1386207323999201001210832 33023441

# Response

**Published:** 2024-02-29

**Authors:** Liliek Susilowati

**Affiliations:** Universitas Airlangga, Indonesia

Thank you for your suggestion.

 I will revise my paper accordingly to your comments and suggestions.

# Reviewer report: Reviewer response for version 1

**DOI:** 10.5256/f1000research.141499.r161413

**Published:** 2023-01-26

**Authors:** Ismail Naci Cangul

**Affiliations:** 1 Department of Mathematics, Bursa Uludag University, Gorukle, Turkey

Set dimension in graph theory is a very applicable area which collected a lot of attention by many people. Here, the authors continued their research with local multiset dimension as a variant and studied this number for amalgamation graphs. Amalgamation is one of the graph operations and this study can easily be carried to other graph operations. I also wondered that this ideas can be applied to algebra as amalgamation plays an important role in algebraic structures. 

 This manuscript is written very clearly as a new step in continuing theory and it will add value to the area. 

 The abstract seems to be uncompiled as there are "$" signs all around. Also Theorems, Propositions, Lemmas must be written in bold to get the attention of the reader.

 Very nice work on multiset theory.

Is the work clearly and accurately presented and does it cite the current literature?

Yes

If applicable, is the statistical analysis and its interpretation appropriate?

Not applicable

Are all the source data underlying the results available to ensure full reproducibility?

No source data required

Is the study design appropriate and is the work technically sound?

Yes

Are the conclusions drawn adequately supported by the results?

Yes

Are sufficient details of methods and analysis provided to allow replication by others?

Yes

Reviewer Expertise:

Graph theory, number theory, discrete group theory, Molecular graphs

I confirm that I have read this submission and believe that I have an appropriate level of expertise to confirm that it is of an acceptable scientific standard.

# Response

**Published:** 2023-01-27

**Authors:** Liliek Susilowati

**Affiliations:** Universitas Airlangga, Indonesia

Dear Prof Ismail,

 For the abstract as there are "$" signs all around. The symbol "$", only editors can fix or remove the "$" sign. I've reported to the editor to remove the "$" sign.

 Best Regards

 Dr. Liliek Susilowati and Dr. Ridho Alfarisi
